# Breeding, genetics, and physiology of improving phosphorus use efficiency in food legumes

**DOI:** 10.3389/fpls.2026.1934844

**Published:** 2026-10-15

**Authors:** Lalchand, Rajesh Ramasandra Venkataraja, Pramod Kumar Meghwal, Ritabrata Sarkar, Deblina Pal, Bhavana Nagenahalli Lakshminarayanaraju, Avinash Pandey, Shambhu Krishan Lal, Vijai Pal Bhadana, Jitendra Kumar, Debjyoti Sen Gupta, Ivica Djalovic, Sudhir Kumar

**Affiliations:** 1 ICAR—Indian Institute of Agricultural Biotechnology, Ranchi, India; 2 ICAR—Indian Institute of Pulses Research, Kanpur, India; 3 Institute of Field and Vegetable Crops Serbia, Novi Sad, Serbia

**Keywords:** genetic variability, GWAS, microbial interactions, omics, PAE, PUE, root architecture, root exudates

## Abstract

Phosphorus (P) is a crucial plant macronutrient, but the non-renewable nature of P sources for plant growth in soils globally limits crop productivity, causing great concern for future sustainable agriculture and global food security. Thus, improving plant phosphorus use efficiency (PUE) by increasing P-utilization and P-acquisition efficiencies is immediately needed in various crops, including legumes, for intensive cropping systems. This review explores how to harness genetic variability in PUE traits across grain legume gene pools using various conventional breeding and emerging breeding approaches. The role of phytohormones in root architecture development, importance of root exudates secretion, and microbe-assisted breeding represent important strategies for improving PUE, considering the non-renewable nature of P resources and its essential role in plant growth. The genetic makeup of PUE is intricate, orchestrated by quantitative traits and significantly affected by environmental factors. Biparental quantitative trait locus (QTL) mapping has been employed to decipher the genetic basis of PUE traits. Progress in legume genomics, particularly high-throughput single nucleotide polymorphism (SNP) genotyping, has enabled genome-wide identification of regions associated with PUE. Concurrently, advancements in multi-omics approaches (functional genomics, transcriptomics, metabolomics and proteomics) have facilitated the identification of key genes, transcription factors and pathways regulating PUE, P transport, recycling and low-P tolerance. This study also elucidated gene networks, signaling pathways, and molecular mechanisms governing P acquisition and utilization essential for improving PUE. We also evaluate the carbon and metabolic costs associated with P-acquisition traits and critically synthesize consistent findings, discrepancies and knowledge gaps across studies. Lastly, we summarize how to formulate innovative breeding strategies, including genomic selection, accelerated breeding, rhizosphere engineering and emerging clustered regularly interspaced short palindromic repeats/CRISPR-associated protein 9 (CRISPR/Cas9)-based genome editing technologies. This may aid in developing P-use-efficient cultivars and adapting grain legumes to low-P environments. Consequently, enhancing PUE by integrating various “omics” approaches could boost P-acquisition and P-utilization efficiencies in modern grain legume cultivars in intensive cropping systems, thereby mitigating P-fertilizer overuse and safeguarding the diminishing non-renewable global rock phosphate resources for ensuring a sustainable and economically viable agrarian future.

## Graphical Abstract

**Figure:**
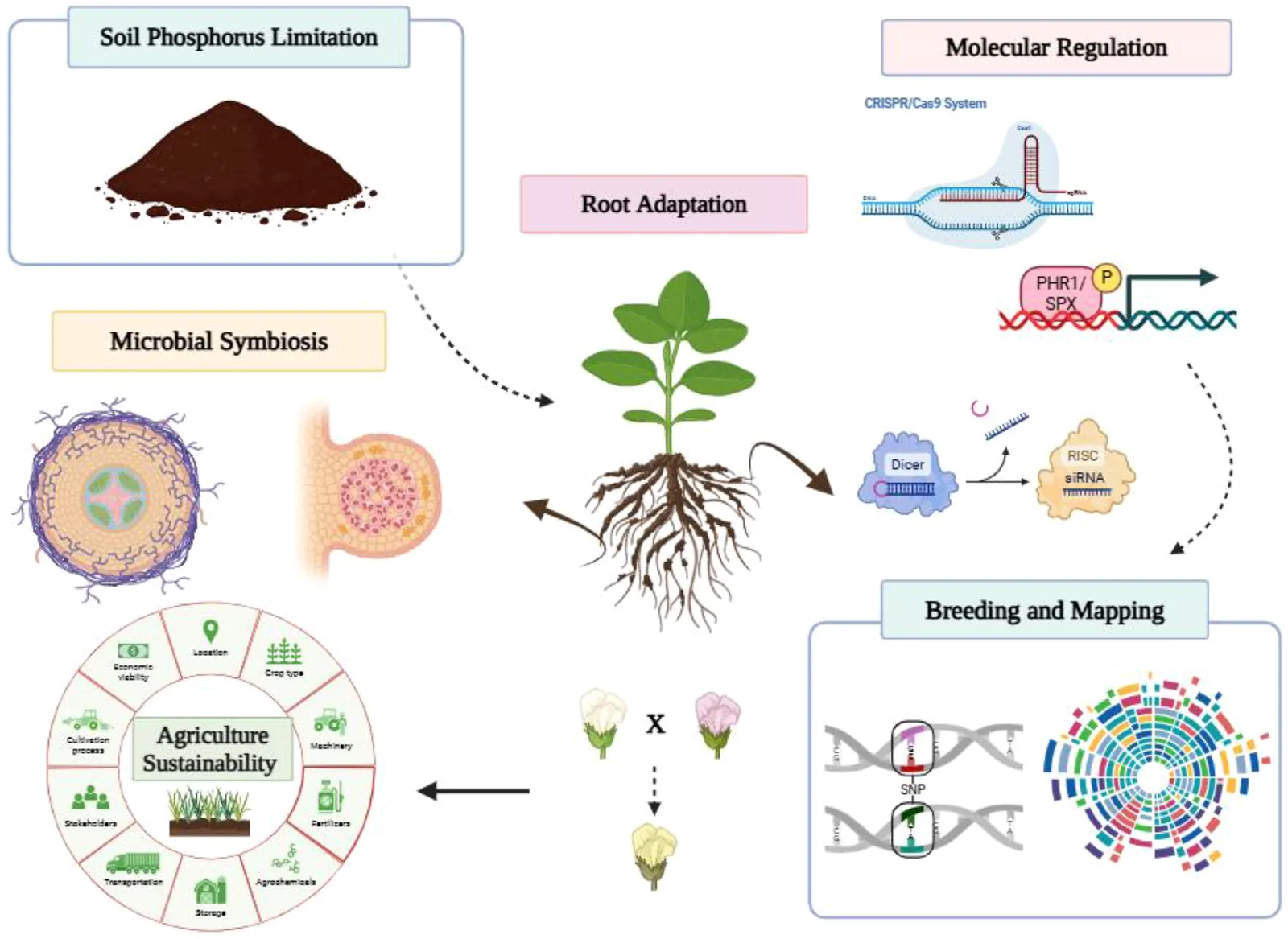
Improving phosphorus use efficiency in legumes under soil phosphorus limitation via root adaptations, microbial symbiosis, and modern breeding approaches to achieve sustainable agricultural productivity.

## 1 Introduction

The element phosphorus (P) is crucial for various biochemical and physiological processes such as respiration, photosynthesis, nucleic acid synthesis, energy generation, and nitrogen fixation in plants (Lambers, 2022). Also plays an important role in seed germination, root growth, flowering, and fruit formation (Malhotra et al., 2018). However, its low availability in soil, especially in inorganic phosphate (Pi) forms (H_2_PO_4_^-^ or HPO_4_²^-^), significantly constrains plant growth and yield across 40%–60% of global arable land (Gutiérrez-Alanís et al., 2018). Significant reductions in photosynthetic rate, stomatal conductance, transpiration rate, and internal CO_2_ concentration were observed during reproductive stages of crop growth (Veronica et al., 2017).

A large percentage (approximately 80%) of applied P fertilizers like diammonium phosphate (DAP), single superphosphate (SSP) and triple superphosphate (TSP) become unavailable to plants due to poor mobility and complex fixation in soils, particularly in acidic soils where phosphate is fixed by Mn²^+^, Al³^+^, and Fe³^+^ (Margenot et al., 2016; Pang et al., 2021; Ojeda-Rivera et al., 2022). This leads to environmental issues like eutrophication from excess application (Zak et al., 2018; Srivastava et al., 2025). Furthermore, the non-renewable nature of rock phosphate, the primary source of P fertilizer (approximately 50 million tons used annually), emphasizes the urgent need for sustainable solutions, as reserves are expected to be depleted in the next 100 years (Bindraban et al., 2020).

Phosphorus-use efficiency (PUE) can be improved through two complementary components: P-acquisition efficiency (PAE; also referred to as P-uptake efficiency, PUpE), which is the ability of plants to take up P from the soil, and P-utilization efficiency (PUtE), which is the ability of plants to convert absorbed P into biomass or grain yield (Wang et al., 2010; Dissanayaka et al., 2018). Past efforts have largely concentrated on the acquisition component, including modifications in root architecture (e.g., more lateral branches and greater root density) (Jia et al., 2018; Wang et al., 2019), increased root exudation of organic anions, protons and enzymes that promote P solubilization (Robles-Aguilar et al., 2019; Wen et al., 2019), and symbiosis with mycorrhizal fungi and phosphate-solubilizing bacteria (Zhang et al., 2016). In comparison, internal PUtE, which depends on P remobilization, P recycling and metabolic P-saving strategies, has received less attention and is therefore also addressed in this review (Van De Wiel et al., 2016; Veneklaas et al., 2012).

This review focuses on the Fabaceae, including major food legumes such as soybean (*Glycine max*), common bean (*Phaseolus vulgaris*), chickpea (*Cicer arietinum*), cowpea (*Vigna unguiculata*), mung bean (*Vigna radiata*), lentil (*Lens culinaris*), faba bean (*Vicia faba*), pea (*Pisum sativum*), pigeonpea (*Cajanus cajan*), peanut (*Arachis hypogaea*), and white lupin (*Lupinus albus*), as well as the model legume *Medicago truncatula* and the tropical forage legume *Stylosanthes guianensis*. Food legumes are vital for global food security and sustainable agriculture because of their ability to fix atmospheric nitrogen (Yang et al., 2022). However, P deficiency reduces legume growth and yield by 30%–40%, largely because nitrogen-fixing root nodules are strong P sinks (Qin et al., 2012; Guo et al., 2022). The review moves from the physiological responses of legumes to P deficiency, to their genetic control, and finally to their use in breeding. *Sections 2–4* describe how root architecture, root exudation and microbial symbioses contribute to P acquisition, and *Section 5* examines the carbon and metabolic costs that these responses impose. *Section 6* evaluates the genetic variation for P-efficiency traits in legume germplasm, and *Section 7* addresses the molecular basis of this variation, covering P-sensing and signaling genes, phosphate transporters, and transcriptomic, proteomic and metabolomic insights. *Section 8* summarizes quantitative trait locus (QTL) mapping and genome-wide association studies (GWAS), and *Section 9* discusses how this knowledge can be translated into P-efficient cultivars. Finally, *Section 10* provides a critical synthesis of consistent findings, discrepancies and knowledge gaps.

## 2 Root architecture of legumes under P-deficient soil

Because Pi is poorly mobile in soil, the first line of plant adaptation to P deficiency is a change in where and how roots grow. All plants exhibit specific morphological changes in response to a lack of individual nutrients. In the majority of natural soils, P is available to plants, but it is mainly in organic forms (like animal matter or dead plant) present in the top layer of soil. P levels decrease in deeper soil layers and that reduces its availability to plant roots (Lynch and Brown, 2001). For efficient absorption of P, root traits are important (Lu et al., 2024). Under P-deficient conditions, plants transport the maximum amount of their photosynthates (sucrose and glucose) to the root, which increases root growth and helps roots explore more soil to absorb P (Nielsen et al., 2001). The root architectural traits associated with improved topsoil foraging include a higher number of adventitious root, shallower basal roots, and root etiolation (under limited-P conditions, the primary root grows less in length, while secondary roots become thicker and spread outwards) (**Figure 1**). This change in root growth is called root etiolation and wider spreading of lateral roots (Lynch, 2011).

**Figure 1:**
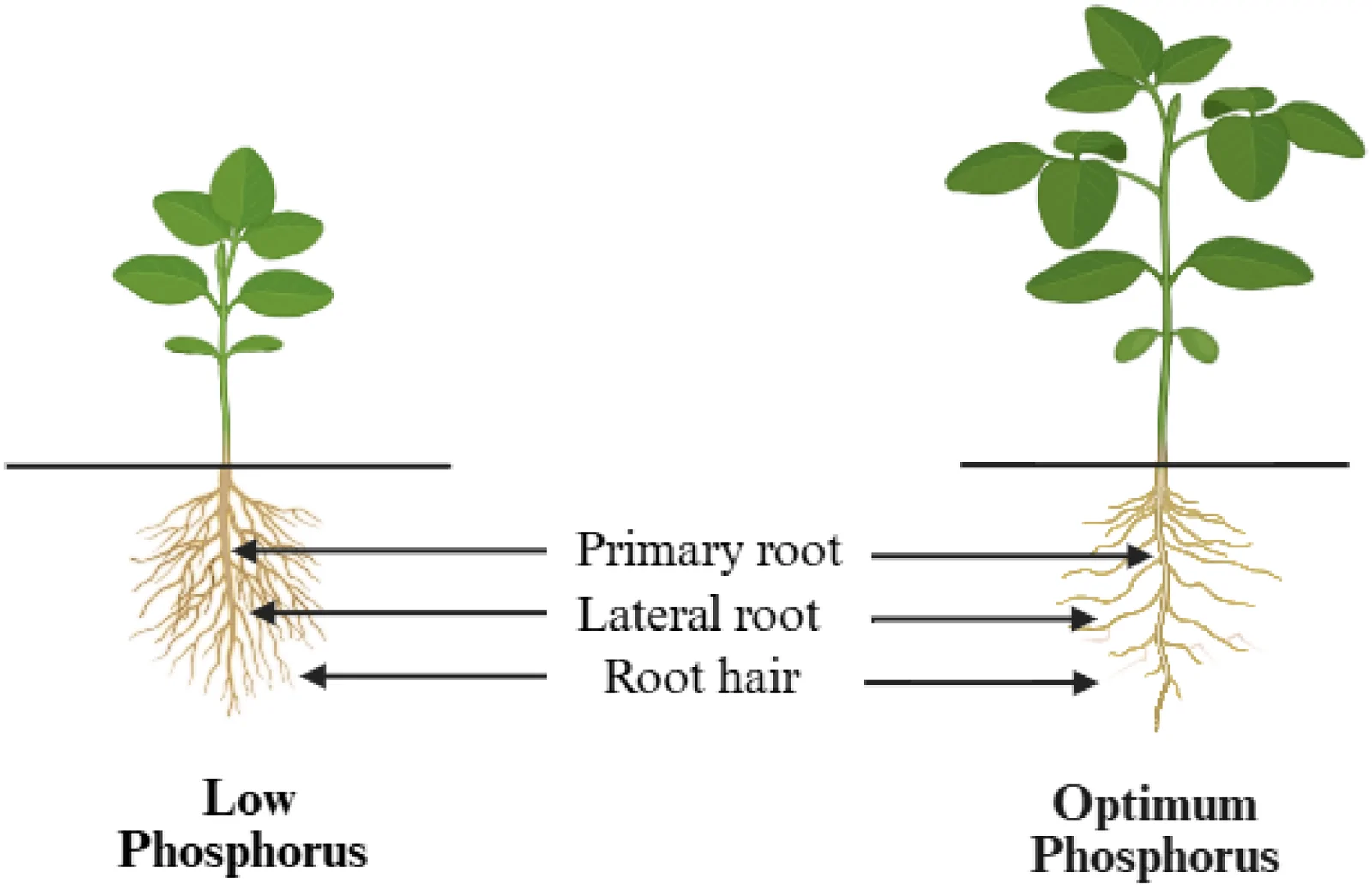
Root architectural responses under different phosphorus availability conditions.

Auxin plays a crucial role in the formation of secondary roots, root hair elongation and the regulation of root hair development (Ma et al., 2001). Ethylene acts in the arrangement of root epidermal cells and the formation of root hairs (Casson and Lindsey, 2003). Reduced cytokinin levels in the root, inhibits the primary root growth and stimulate shoot growth (Martín et al., 2000). Strigolactones, another class of phytohormones, regulate lateral root formation and root hair elongation under limited-P conditions, as shown in *Arabidopsis thaliana* (**Table 1**) (Mayzlish-Gati et al., 2012).

**Table 1:** Role of phytohormones in root system development of legumes under low-P conditions.

Phytohormone	Role in root physiology	Crop	References
Auxin	Low-P increases auxin accumulation in roots, stimulating lateral root formation and root hair elongation, increasing soil exploration for P acquisition	Soybean, common bean	Sun et al. (2015)
Ethylene	Regulates root hair formation and interacts with auxin to modify root architecture	Common bean	Ma et al. (2001); Sun et al. (2015)
Cytokinin	Generally, suppresses root growth, lower cytokinin levels, promote root elongation and branching	Soybean	Sun et al. (2015)
Gibberellins	Modulate primary root elongation and overall root growth	Chickpea, soybean	Sun et al. (2015)
Abscisic Acid	ABA helps maintain root growth and improves nutrient uptake efficiency, enhances stress tolerance	Peanut, soybean	Hernández et al. (2007)
Strigolactones	Stimulate root hair growth and enhance symbiotic association with mycorrhiza for improved P uptake	Pea, soybean	Sun et al. (2015)

Modifications in root structure under low-P conditions have been well documented in legumes such as chickpea (*C. arietinum*) (Singh et al., 2015), soybean (*G. max*) (Ao et al., 2010), and common bean (*P. vulgaris*) (Lazali et al., 2018). Soybean plants showed reduced primary root growth and increased development of secondary roots and root hairs (Zhu et al., 2020). Common bean plants developed shallow basal roots (Liao et al., 2001). White lupin showed a special root modification by forming cluster roots, and their formation is regulated by phytohormones such as auxin and cytokinin (Neumann et al., 2000). Cluster root formation is one of the highly effective ways plants adapt to increase P acquisition (Lamont, 2003). These roots consist of many short and closely packed rootlets covered with dense root hairs, which are highly effective at releasing and absorbing P from the soil. *Lupinus angustifolius* and *L. albus* also have cluster roots, which effectively utilize soil phytate by enhancing the rhizospheric microorganisms like *Burkholderia* species (Lambers et al., 2013).

The other root architecture system modification that enhances P acquisition is root hair formation, which is usually accompanied by rises in the root-to-shoot ratio. Under low-P conditions, root hairs, cause approximately 90% of the P gain and provide approximately 70% of the total root surface (Lazali and Drevon, 2021). Root hairs help roots explore a larger area of the soil, reach soil pores that the plant cannot access on its own, and release substances into the soil (Lazali and Drevon, 2021).

Overall, the available evidence indicates that changes in root architecture, particularly increased lateral root development, root hair formation, and greater root exploration, can contribute to improved P acquisition under P-deficient conditions. However, the contribution of individual root traits varies among genotypes and environments, indicating that no single root trait can consistently explain PUE. These root architectural modifications are complemented by changes in the rhizosphere, where root exudation can mobilize P from otherwise unavailable soil pools and further enhance P acquisition.

## 3 Role of legume root exudates under low-P soil

Root architecture determines the volume of soil that is explored, but much of the P within that volume is bound in forms that roots cannot take up directly. Legumes therefore complement root growth with the release of P-mobilizing compounds into the rhizosphere. Soil P exists as inorganic forms (calcium, iron, and aluminum phosphates) that are largely unavailable to plants (Ao et al., 2010), and available organic forms (nucleotides, phospholipids, and phytate) (Simpson et al., 2011). Under soil P limitation, nodulated (N_2_-fixing) common bean plants acidify the root zone by releasing more protons (H^+^), because they take up more cations than anions (Alkama et al., 2012). This enhanced soil acidity helps to solubilize phosphate from insoluble forms, particularly in neutral, calcareous, or rock-phosphate-amended soils (Zhang et al., 2014). Many legumes, including soybean, common bean, white lupin, and chickpea, release P-mobilizing compounds such as phosphatases, organic acids, and other root exudates, which convert unavailable P into plant-accessible forms (Dong et al., 2004; Lazali and Drevon, 2021).

Low-molecular-weight organic acids (carboxylates) play a role by chelating metal ions (Al³^+^, Ca²^+^, Fe³^+^) that bind P, thus releasing phosphate for uptake (Jones and Oburger, 2011). Common root exudates include acetate, oxalate, citrate, succinate, lactate, and malate. For instance, white lupin cluster roots release citrate equivalent to approximately 23% of their dry biomass (Lambers et al., 2008), making *L. albus* a model for organic acid studies. Organic acid-mediated P mobilization also enhances aluminum tolerance but depends on soil mineral composition, acid concentration, and pH (Hinsinger et al., 2015). These acids are short-lived in soil due to adsorption or microbial degradation (Wang et al., 2010). Although increased carboxylate and organic acid exudation can enhance P mobilization from poorly available soil P pools, greater exudation does not necessarily result in higher PUE or grain yield. This indicates that the effectiveness of root exudation depends on genotype, soil properties, P source, and the plant’s capacity to utilize the mobilized P, and is further influenced by rhizosphere microorganisms that enhance P solubilization and mineralization, thereby complementing plant-mediated P acquisition.

## 4 Role of microbes in enhancing PUE of legumes

Root exudates also shape the rhizosphere microbiome, and legumes further extend their P-acquisition capacity through associations with soil microorganisms. Beneficial soil microbes (*Bacillus*, *Pseudomonas*, *Rhizobium*, *Enterobacter*, and *Glomus*) enhance plant nutrient uptake and help them cope with both biotic and abiotic stresses (Jat et al., 2021; Meghwal et al., 2026). Phosphorus-solubilizing microorganisms (PSMs) significantly contribute to enhancing the availability of P for legumes, which is essential for their growth and productivity, especially for biological nitrogen fixation (Janati et al., 2021). Although P is abundant in soils, a significant portion is typically unavailable to plants because it is fixed in insoluble organic and inorganic forms through chelation with metal ions including iron, calcium, and aluminum (Gurav et al., 2024; Iftikhar et al., 2024; Bashir et al., 2024; Rawat et al., 2021). This unavailability makes P a major limiting nutrient for plant growth globally (Kalayu, 2019; Yingjie et al., 2023). PSMs, including diverse bacteria, actinomycetes and fungi, convert these insoluble phosphate forms into soluble and plant-available forms via several mechanisms, hence acting as eco-friendly biofertilizers (Santoyo et al., 2025; Wang et al., 2023a; Fu et al., 2024; Timofeeva et al., 2022). The key mechanisms employed by PSMs for phosphate solubilization include the release of inorganic and organic acids, the production of enzymes like phosphatases and metal-chelating molecules (Kumar et al., 2020; Li et al., 2022a). Organic acids, like lactic acid, citric acid, and gluconic acid, secreted by PSMs can chelate cations that bind to phosphate, hence releasing Pi into the soil solution (Iftikhar et al., 2024).

Microbe-assisted breeding represents an important strategy for improving PUE, considering the non-renewable nature of P resources and its essential role as a macronutrient in plant growth. This approach harnesses beneficial plant–microbe interactions to enhance P acquisition and utilization (Bargaz et al., 2021). The symbiotic interaction between soil microbes and legumes, particularly arbuscular mycorrhizal fungi and rhizobia, plays a central role in the improvement of PUE. Rhizobia are well known for establishing nitrogen-fixing symbiosis within root nodules, which is important for soil fertility and legume growth (Liu et al., 2020; Muthini et al., 2020). However, this process is highly dependent on P, and low-P availability can significantly reduce nodule formation and activity, thereby limiting nitrogen fixation (Liu et al., 2024b). Legumes have developed advanced molecular mechanisms that allow them to interact with beneficial soil microbes (**Figure 2**), which help plants to take up more nutrients efficiently (Zhao et al., 2023; Feki et al., 2020).

**Figure 2:**
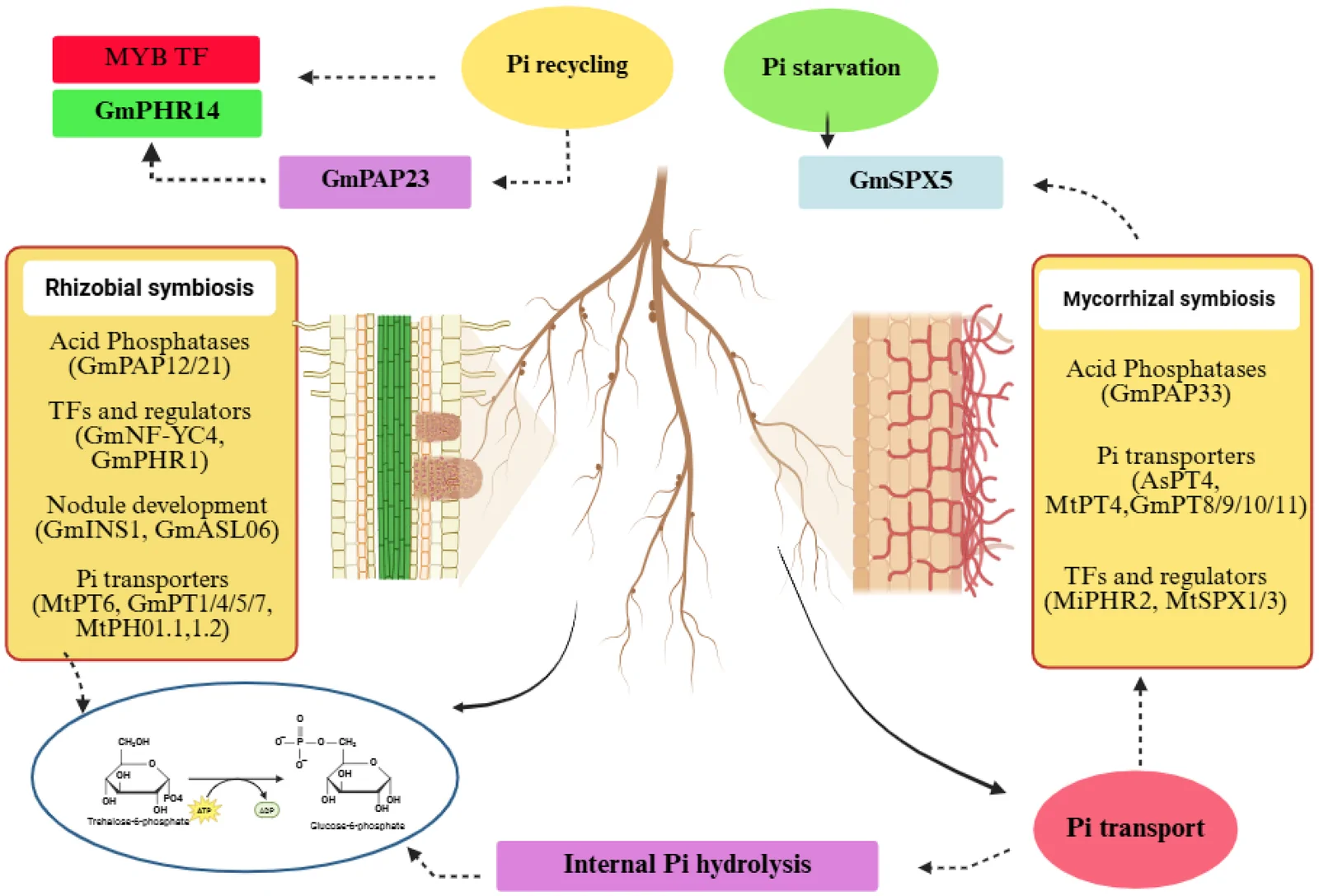
Schematic representation of inorganic phosphate (Pi) acquisition, transport, and homeostasis in plant roots under low-P conditions. It highlights regulatory genes, transcription factors, and symbiotic interactions (rhizobial and mycorrhizal) that enhance Pi uptake and availability.

The formation of these symbioses is regulated by complex chemical signaling (Singh et al., 2025a). For example, plants initiate AM symbiosis by releasing chemical signals such as strigolactones from their roots under low-P conditions (Li et al., 2023; Lambers, 2022). Strigolactones serve as an attractant for AM fungal spores, signaling the plant’s need for phosphate and stimulating fungal germination and growth towards the root. Additionally, this single chemical signal coordinates the plant’s internal resource allocation by inhibiting shoot branching to conserve resources. Once recognized, AM fungi develop complex structures called arbuscules within the plant root cells, dramatically increasing the surface area available for nutrient exchange (Mitra et al., 2023). The fungi extend fine hyphae deep into the soil, covering a larger volume than plant roots alone, which is critical for acquiring immobile nutrients such as phosphate. The fungus takes up phosphate ions, converts them into polyphosphate for long-distance transport, then releases Pi into the periarbuscular space, where it is absorbed by plant cells through specialized transporters. Various genes participate in the regulation of phosphate uptake and transport in legumes, many of which are regulated at the time of symbiotic interactions. *GmAGL82*, a MADS-box transcription factor in *G. max*, is preferentially expressed in nodules and exhibits increased expression under low-P conditions. Overexpression of this gene promotes the growth of root nodules, indicating its involvement in associating nodulation with P availability (Song et al., 2024). Genes of the nodulation signaling pathway, such as *NSP1* and *NSP2*, also regulate the expression of iron, sulfur and P transporter genes, hence influencing P accumulation and vanadium tolerance in legumes depending on rhizobial symbiosis and the rhizosphere microbiome (Liu et al., 2024a). This highlights the interconnected nature of nutrient signaling pathways. The common symbiosis signaling pathway (CSSP) is essential for establishing both nitrogen-fixing nodulation and AM symbiosis in legumes (Singh et al., 2025a). Important genes in this pathway, like those encoding a calcium-dependent kinase called CCaMK, are required for the establishment of both symbioses. The CSSP evolved to perceive microbial signals, like strigolactones from AM fungi and nod factors from rhizobia, and to translate them into calcium oscillations that activate symbiotic gene expression. This pathway indicates an ancient evolutionary innovation that legumes have co-opted to support nitrogen fixation. Auxin/indole-acetic acids (AUX/IAAs) and auxin response factors (ARFs) involved in the auxin signaling pathway co-regulate the genes that are responsible for transcription of auxin. For example, the *SlIAA23-SlARF6* module regulates AM symbiosis in tomato (*Solanum lycopersicum*) by controlling biosynthesis of strigolactone (Li et al., 2023). This emphasizes the role of hormonal regulation in plant–microbe interactions that are essential for P uptake.

Taken together, AM fungi, rhizobia, and phosphate-solubilizing microorganisms extend the P-acquisition capacity of legumes well beyond what roots achieve alone. A consistent finding across studies is that the benefit of these associations is greatest in low-P soils, whereas the magnitude of the benefit depends strongly on the microbial strain or fungal isolate, the plant genotype, and soil conditions (Richardson et al., 2011; Wang et al., 2016). Inoculation effects observed under controlled conditions are also frequently smaller or less consistent in the field. Like root proliferation and exudation, these microbial partnerships require carbon from the host, and their net value for PUE therefore depends on the balance between the P gained and the carbon invested, as discussed in the next section.

## 5 Physiological trade-offs associated with improving PUE

Many of the adaptive responses described in Sections 2–4 increase P acquisition, but they are not free of cost. Root proliferation, cluster-root formation, carboxylate exudation, phosphatase secretion, and the maintenance of mycorrhizal and rhizobial symbionts all consume photosynthetically fixed carbon and other resources that could otherwise be allocated to shoot growth and grain filling (Lynch and Ho, 2005; Lambers, 2022). Whether a given trait improves PUE therefore depends on the balance between the additional P it provides and the resources it consumes, and this balance changes with soil type, P availability, water supply, and crop developmental stage.

Root construction and maintenance represent the largest of these costs. Under P deficiency, a considerable fraction of daily photosynthate can be spent on root respiration, and common bean genotypes differ in the amount of carbon they invest in roots per unit of P acquired (Nielsen et al., 2001; Lynch and Ho, 2005). Root phenes that reduce the metabolic cost of soil exploration, such as greater root hair length and density and more lateral branching in the topsoil, can therefore improve P acquisition more efficiently than a simple increase in total root biomass (Lynch, 2011). Root hairs are a particularly economical investment because they substantially increase the absorptive surface at a small carbon cost (Lazali and Drevon, 2021). By contrast, a greater root-to-shoot ratio can improve P uptake while reducing the proportion of assimilates available for shoot growth and yield, which is one reason why larger root systems do not consistently translate into higher grain yield.

Carboxylate exudation and mycorrhizal symbiosis represent alternative, partly substitutable investments of carbon. In a comparison of 16 crop species, Wen et al. (2019) found that root diameter was associated with contrasting P-acquisition strategies: thin-rooted species tended to invest in root proliferation, whereas thick-rooted species relied more on mycorrhizal symbiosis, indicating trade-offs among root morphology, exudation, and mycorrhizal strategies. Similarly, chickpea genotypes differing in rhizosheath carboxylate amounts showed contrasting patterns of biomass allocation, root morphology, and mycorrhizal colonization (Wen et al., 2020), and a negative relationship between rhizosphere carboxylates and arbuscular mycorrhizal colonization has been reported among legumes (Ryan et al., 2012). White lupin represents the extreme case, combining very high carboxylate release from cluster roots with a non-mycorrhizal habit (Lambers et al., 2013). These studies suggest that breeding programs should not attempt to maximize every acquisition trait simultaneously, but should select combinations of traits that suit the target soil.

In legumes, an additional trade-off arises from symbiotic nitrogen fixation. Nodules have a high P requirement and act as a strong P sink; under P deficiency, nodule number, nodule mass, and nitrogen fixation decline, and nodulated-root respiration increases the carbon cost of nitrogen acquisition (Qin et al., 2012; Alkama et al., 2012; Lazali and Drevon, 2021). P efficiency in legumes therefore has to be assessed together with nitrogen fixation, because genotypes that conserve P by reducing nodulation may become nitrogen-limited.

A further trade-off concerns the two components of PUE. Improving PAE increases total P uptake, whereas improving PUtE increases the biomass or grain produced per unit of absorbed P, often by lowering tissue P concentration, remobilizing P more efficiently from senescing leaves, or replacing phospholipids with non-phosphorus lipids (Wang et al., 2010; Veneklaas et al., 2012). The relative importance of the two components depends on the production system: acquisition traits are generally more valuable where soil P is strongly fixed, whereas utilization traits are more relevant where fertilizer inputs are to be reduced without yield loss (Wang et al., 2010). Improvements in PUtE can also carry costs. Lower grain P concentration reduces the removal of P from the field and can lower phytate, which may improve the bioavailability of iron and zinc; however, extremely low seed P can impair seedling vigor and establishment on P-deficient soils (White and Veneklaas, 2012). Likewise, excessive remobilization of P from leaves may accelerate senescence and reduce photosynthesis during the reproductive stage (Veneklaas et al., 2012; Veronica et al., 2017).

Finally, the value of a given acquisition trait depends on co-occurring stresses. A shallow root system that favors topsoil foraging for P can limit access to subsoil water under drought (Lynch, 2011). In chickpea, P acquisition under a drying topsoil depends on subsoil root activity and rhizosheath carboxylates (Kabir et al., 2015; Sharma et al., 2021), as well as on leaf transpiration, which drives the mass flow of P towards the root surface (Pang et al., 2018a). Similarly, the effectiveness of exuded carboxylates depends on soil mineralogy, pH and microbial degradation (Jones and Oburger, 2011; Hinsinger et al., 2015). Consequently, the same trait may be beneficial in one environment and neutral or costly in another, which partly explains the inconsistent associations between individual P-acquisition traits and yield reported across studies (*Section 10*). These trade-offs indicate that ideotypes for P efficiency should be defined for specific target environments, and that traits should be evaluated not only for the P they acquire but also for their carbon cost and their effect on yield. Identifying such trait combinations requires genetic variation, the extent of which in legume germplasm is examined in the next section.

## 6 Genetic variation for P-efficiency traits in legume germplasm

The adaptive responses and trade-offs described above are not uniform among genotypes. Significant genetic variation has been observed for PUE in legumes (Singh, 1992; Fageria and Da Costa, 2000; Wen et al., 2020). Crop genotypes vary in their ability to change root architecture systems, such as root length, lateral root formation, root hair density, and root angle, which directly affect P uptake under P-deficiency conditions (Pan et al., 2008; Powers et al., 2020). Genetic variation is also observed in physiological traits like phosphatase activity, organic acid exudation, and mycorrhizal responsiveness, all of which contribute to increased P absorption and mobilization. This genetic variation gives a strong basis for selection and breeding of P-efficient genotypes adapted to P-deficient soils (Wen et al., 2020).

Screening studies in cowpea, faba bean, mung bean, lentil, pea, soybean, common bean, and chickpea (**Table 2**) lead to three general conclusions. First, genetic variation for P-efficiency traits is large in all species examined, and root traits such as root hair length and density, basal root number and taproot length can show high heritability, making them suitable targets for selection (Mohammed et al., 2022; Opoku et al., 2022). Second, genotypes identified as P-efficient differ in the mechanism underlying their efficiency: some combine superior root morphology with high P uptake (e.g., mung bean, lentil, and soybean; Reddy et al., 2020; Aski et al., 2022; Zhou et al., 2016), some rely on carboxylate or malate exudation (chickpea and soybean; Pang et al., 2018b; Liang et al., 2013; Reddy et al., 2021b), and others on efficient internal P utilization and remobilization to grain (faba bean and pea; Daoui et al., 2012; Powers et al., 2020). Third, genotype rankings are frequently unstable across P levels, water regimes and sites. In faba bean, for example, one variety performed best under moderate P availability owing to efficient P utilization and assimilate partitioning to grain, whereas another was superior under severe P deficiency and higher rainfall (Daoui et al., 2012), and several cowpea genotypes maintained biomass under low P but did not respond to additional P (Opoku et al., 2022). The relative contribution of P acquisition and P utilization to overall PUE therefore varies with genotype and environment (Brahimi et al., 2023).

**Table 2:** List of legume genotypes with efficient P uptake and utilization capacity.

Crop	Genotype name	Place	Number of genotypes	Key findings	References
Cowpea	Agyenkwa, Nketewadea, Songotra, Secow 2 W, Secow 5 T	Minjibir, Kano, Zaria in Nigeria	60	Agyenkwa and Nketewadea showed high root dry weight, shoot dry weight, root hair length and root hair density. Secow 5 T and Secow 2 W had the high P-uptake efficiency, PUE.	Opoku et al. (2022)
Cowpea	TVu-7778, Vita7, Sanzi, IT98K-111-1, IT96D-610, IT97K-499-35	University of California, Riverside (UCR) and Apache Root Biology Center (ARBC) Arizona, both in the United States.	50	Long tap root, high root hair. Sanzi, TVu-7778 had high lateral root hair density. Vita7, IT97K-499–35 had high tap root density.	Mohammed et al. (2022)
Faba bean	Sidi Aich, Castel, Diva	Haut Cheliff plain, Algeria	3	Shoot dry weight, Root dry weight, Castel showed high P concentration in seed, root, shoot, nodules. Diva had higher PUE	Brahimi et al. (2023)
Faba bean	Defes	National Institute of Agronomic Research of Morocco (INRA-Morocco)	4	P-harvest index, had higher yield and PUE than Karabiga, Lobab and Aguadulce	Daoui et al. (2012)
Mung bean	IPM-288, TM96-2, TM 96-25, PUSA 1342, M 1477	Indian Agricultural Research Institute (IARI), New Delhi, India.	153	Root length, total root volume, total root surface area	Reddy et al. (2020)
Mung bean	PUSA 1333, Pusa Vishal, Pusa Ratna, and PUSA 1031	IARI, New Delhi	36	Root dry weight, root to shoot ratio, P- utilization efficiency	Reddy et al. (2021b)
Lentil	L4727, EC714238, EC718309, EC718348, PL-97, EC18332, DPL15, PLO6	IARI, New Delhi	85	Total dry weights, shoot dry weight, root dry weight.	Ramtekey et al. (2021)
Lentil	IC 560135, IG-Y-50, IG 334	IARI, New Delhi	40	Total root length, total surface area, root average diameter. IG-Y-50 had higher root dry weight, shoot dry weight, total dry weight. IG 334 had higher p-uptake efficiency, p-utilization efficiency.	Aski et al. (2020)
Lentil	PLL 18-09, PLS 18-01, PLL 18-25, PLS 18-23, and PLL 18-07	IARI, New Delhi	110	Total root length, primary root length, root average diameter, total root surface area, total root forks, total root tips, total root volume	Aski et al. (2022)
Pea	PI 413683 and PI411142	-	50	Enhanced phosphorus remobilization	Powers et al. (2020)
Soybean	E311 and E141	Sichuan Agricultural University in Ya’an, Sichuan province, China	274	Specific root length, root surface area, root volume and total root length	Zhou et al. (2016)
Soybean	HN89	–	2	Shallow rooted architecture, increased malate exudation	Liang et al. (2013)
Common bean	Carioca comum, IAPAR 81, IAC Imperador, G2333	Campinas, in the state of Saã;o Paulo, Brazil	20	Root surface area and root length	Silva et al. (2014)
Chickpea	ICC456	University of Western Australia, Australia	2	Shoot and root traits, yield and yield component	Pang et al. (2022)
Chickpea	ICC 5504, ICC 8318, ICC 4872, ICC 16796	The University of Western Australia, Australia	100	Root dry weight, total carboxylates per plant	Pang et al. (2018b)
Chickpea	Go¨kc e’ and ‘C agatay’	C ukurova University, Faculty of Agriculture, Department of Soil Science and Plant Nutrition	9	Under low phosphorus conditions, Gökçe and Çağatay recorded higher shoot dry biomass.	Gülüt and Özdemir (2021)

The main limitation of this body of work is methodological. Most screenings were carried out at the seedling stage in pots, hydroponics or rhizotrons, used different definitions of PUE (e.g., biomass per unit P uptake, grain yield per unit P applied, or relative performance under low versus high P), and rarely related seedling traits to grain yield in the field (Aski et al., 2020; Reddy et al., 2021a; Ramtekey et al., 2021). Consequently, results are difficult to compare across studies, and it remains uncertain whether genotypes that are efficient at the seedling stage remain efficient at maturity, when a large part of P uptake occurs (Wang et al., 2022). Standardized PUE definitions and multi-environment field evaluation of the promising genotypes listed in **Table 2** are therefore needed before they can be used with confidence as donor parents.

Overall, substantial genotypic variation exists for P acquisition, P utilization, root traits, biomass, and yield under P-deficient conditions. However, the performance of P-efficient genotypes may vary with P availability and environmental conditions, highlighting the importance of selecting genotypes based on a combination of root, physiological, P efficiency, and yield-related traits across multiple environments. Such genetic variation provides a foundation for breeding P-efficient legume genotypes. However, understanding the molecular mechanisms underlying this variation is essential for identifying functional genes and regulatory pathways controlling P acquisition and utilization.

## 7 Molecular basis of P sensing, transport and utilization in legumes

The genetic variation described above ultimately reflects differences in genes that sense P status, transport Pi and adjust metabolism to P supply. This section first describes the key gene families involved in P sensing, signaling, and root development (*Section 7.1*) and the phosphate transporters that move Pi into and within the plant (*Section 7.2*). It then summarizes transcriptomic and proteomic (*Section 7.3*) and metabolomic (*Section 7.4*) studies that show how these genes are coordinated during P starvation. The main genes are listed in **Table 3** and illustrated in **Figures 3**–**5**.

**Table 3:** Key genes involved in PUE and PAE in legume species.

Crop	Gene	Role in P acquisition/use efficiency	References
*G. max*	*GmEXPB2* *(Glycine max β-expansin 2)*	Increases primary root elongation by increasing cortical cell size and number, improves Pi uptake and P efficiency	Li et al. (2015)
*G. max*	*GmPAP23 (Glycine max purple acid phosphatase 23)*	Hydrolyzes organic P compounds, release inorganic phosphate and enhance P remobilization.	Guo et al. (2025)
*G. max*	*GmPT2 (Glycine max Phosphate transporter 2)*	Promotes P uptake from soil via root epidermal and cortical cells, increases root-to-shoot translocation of Pi, supporting plant growth under P-deficient conditions.	Yao et al. (2014a)
*G. max*	*GmPTF1 (Glycine max Phosphate Transcription Factor 1)*	Increases Pi uptake, root adaptation, and PUE.	Yang et al. (2021a)
*G. max*	*GmSPX5 (Glycine max SYG1–PHO81–XPR1 5)*	Increase AM colonization, plant biomass and total phosphorus content.	Yang et al. (2025)
*G. max*	*GmPT5 (Glycine max Phosphate transporter 5)*	Increase PUE by enhancing P uptake and mobilization in plants.	Qin et al. (2012)
*G. max*	*GmPT4 (Glycine max Phosphate Transporter 4)*	Enhance Pi uptake and internal transport, improving PAE.	Guo et al. (2022)
*G. max*	*GmPT7 (Glycine max Phosphate Transporter 7)*	Enhanced nodulation, plant biomass and phosphorus uptake	Chen et al. (2019)
*G. max*	*GmPHO2 (Glycine max Phosphate 2)*	Negative regulator of phosphate transporters, involved in systemic P signaling.	Valdés-López et al. (2008)
*G. max*	*GmETO1 (Glycine max Ethylene Overproducer 1)*	Increases root development, improving P acquisition	Zhang et al. (2020)
*G. max*	*GmPHR25 (Glycine max Phosphate Starvation Response 25)*	Coordinates expression of Pi transporters and PAPs to increase PUE	Xue et al. (2017)
*G. max*	*GmPAP7a (Glycine max Purple Acid Phosphatase 7a)*	Increases root-associated phosphatase activity and organic P utilization	Zhu et al (2020)
*G. max*	*GmPAP14 (Glycine max Purple Acid Phosphatase 14)*	Enhance utilization of phytate and organic P, increasing PUE	Kong et al. (2018)
*G. max*	*Gm6PGDH1 (Glycine max 6-phosphogluconate dehydrogenase 1)*	Increases root development and Pi uptake	Li et al (2021)
*G. max*	*GmMDH12 (Glycine max Malate dehydrogenase 12)*	Promotes plant growth and P uptake	Zhu et al. (2021)
*G. max*	*GmWRKY46 (Glycine max WRKY transcription factor 46)*	Enhance root growth and Pi uptake by activating P-responsive genes	Li et al. (2021b)
*G. max*	*GmEXLB1 (Glycine max Expansin-like B1)*	Enhance PAE by improving root length and surface area	Kong et al. (2019)
*G. max*	*GmPAP7b (Glycine max Purple Acid Phosphatase 7b)*	Increases root-associated phosphatase activity and organic P utilization	Zhu et al (2020)
*G. max*	*GmGDPD2 (Glycine max Glycerophosphodiester Phosphodiesterase 2)*	Improves P efficiency and root architecture	Zuo et al. (2025)
*G. max*	*GmACP1 (Glycine max Acid Phosphatase 1)*	Enhances P availability in the rhizosphere by breaking down organic phosphate esters	Zhang et al. (2014)
*G. max*	*GmPHT1 (Glycine max Phosphate Transporter 1)*	Enhance phosphorus acquisition efficiency and root phosphate absorption capacity	Wei et al. (2022)
*G. max*	*GmPT8/9/10/11 (Glycine max Phosphate Transporter 8)*	Enhances mycorrhizal P uptake pathway, complementing the direct root uptake system and improves PAE	Wang et al. (2016)
*L. albus*	*LaMATE1/3 (Lupinus albus Multidrug and Toxic compound extrusion1/3)*	Enhances solubilization of insoluble phosphorus and Increase PAE	Zhou et al. (2021)
*L. albus*	*LaABCG29 (Lupinus albus ATP-Binding Cassette transporter G, subfamily 29)*	Enhances phosphorus mobilization in the rhizosphere and PAE	Aslam et al (2021)
*L. albus*	*LaCEP1 (Lupinus albus C-terminally encoded peptide 1)*	Negatively regulates cluster root development, indirectly regulates PAE	Zhou et al. (2019)
*L. albus*	*LaABCG37s (Lupinus albus ATP-Binding Cassette Transporter G, Subfamily 37)*	Enhances solubilization of insoluble P forms, cluster root formation and improve PAE	Xu et al. (2020)
*L. albus*	*LaGPX-PDE1/2 (Lupinus albus Glycerophosphodiester phosphodiesterase 1/2)*	Essential for the root hair development and elongation	Cheng et al. (2011)
*L. albus*	*LaPHT1 (Lupinus albus Phosphate Transporter 1)*	Regulates the expression of genes involved in P acquisition and metabolism under P deficiency	Aslam et al. (2021a)
*P. vulgaris*	*PvSPX1 (Phaseolus vulgaris SPX domain-containing protein 1)*	Modulates P signaling pathways, often interacting with PHR1	Yao et al. (2014b)
*P. vulgaris*	*PvPAP3 (Phaseolus vulgaris Purple Acid Phosphatase 3)*	Localizes to apoplast; enhances utilization of external organic P	Liang et al. (2010)
*P. vulgaris*	*PvPHR1 (Phaseolus vulgaris Phosphate Starvation Response 1)*	Regulates the expression of genes involved in P acquisition and metabolism under low-P	Valdés-López et al. (2008)
*P. vulgaris*	*PvPHT1 (Phaseolus vulgaris Phosphate Transporter 1)*	Uptake of Pi from soil and its translocation within the plant	Cao et al. (2018)
*C. arietinum*	*CaPHO1 (Cicer arietinum PHOSPHATE 1)*	Root to shoot Pi export and nodule development. Responsible for loading Pi into xylem parenchyma cells of the roots.	Mani et al. (2024)
*M. truncatula*	*MtPT4 (Medicago truncatula Phosphate Transporter 4)*	Specialized *PHT1* family transporter exclusively expressed in arbuscule-containing cells for symbiotic P uptake from AM fungi.	Fellbaum et al. (2014)
*M. truncatula*	*MtPT6 (Medicago truncatula Phosphate Transporter 6)*	Enhance PAE of plants.	Cao et al. (2021)
*M. truncatula*	*MtPHO2 (Medicago truncatula Phosphate 2)*	Regulating the root to shoot translocation of Pi and involve in nodule formation.	Huertas et al. (2023)
*S. guianensis*	*SgEXPB1 (Stylosanthes guianensis β-expansin 1)*	Increased Pi uptake through improved root elongation.	Wang et al. (2023b)

**Figure 3:**
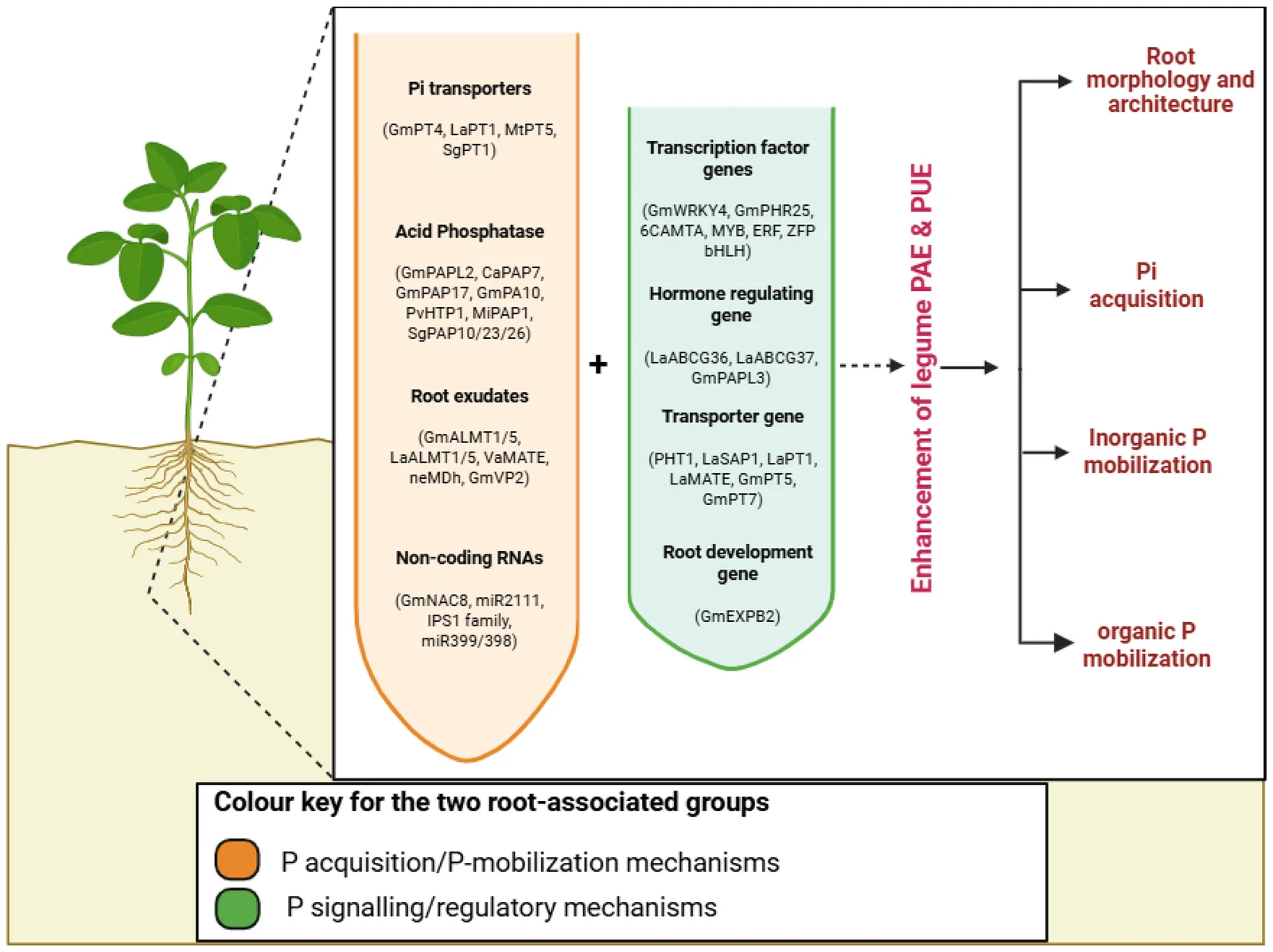
Overview of genes regulating P acquisition and utilization efficiency in legumes. The orange (left) panel groups effector genes that act directly on P acquisition, namely Pi transporters, acid phosphatases, genes controlling root exudation and P-responsive non-coding RNAs. The green (right) panel groups regulatory genes, namely transcription factors and hormone-related genes, together with genes controlling root growth, which modulate the expression or activity of these effectors. The two panels are drawn as stylized root tips for illustration only and do not represent different root types or tissues. Together, the two gene groups enhance PAE and PUE through changes in root morphology and architecture, Pi acquisition, and the mobilization of inorganic and organic P. Phosphate transporters and P-saving metabolic pathways are shown in more detail in **Figures 4**, **5**.

**Figure 4:**
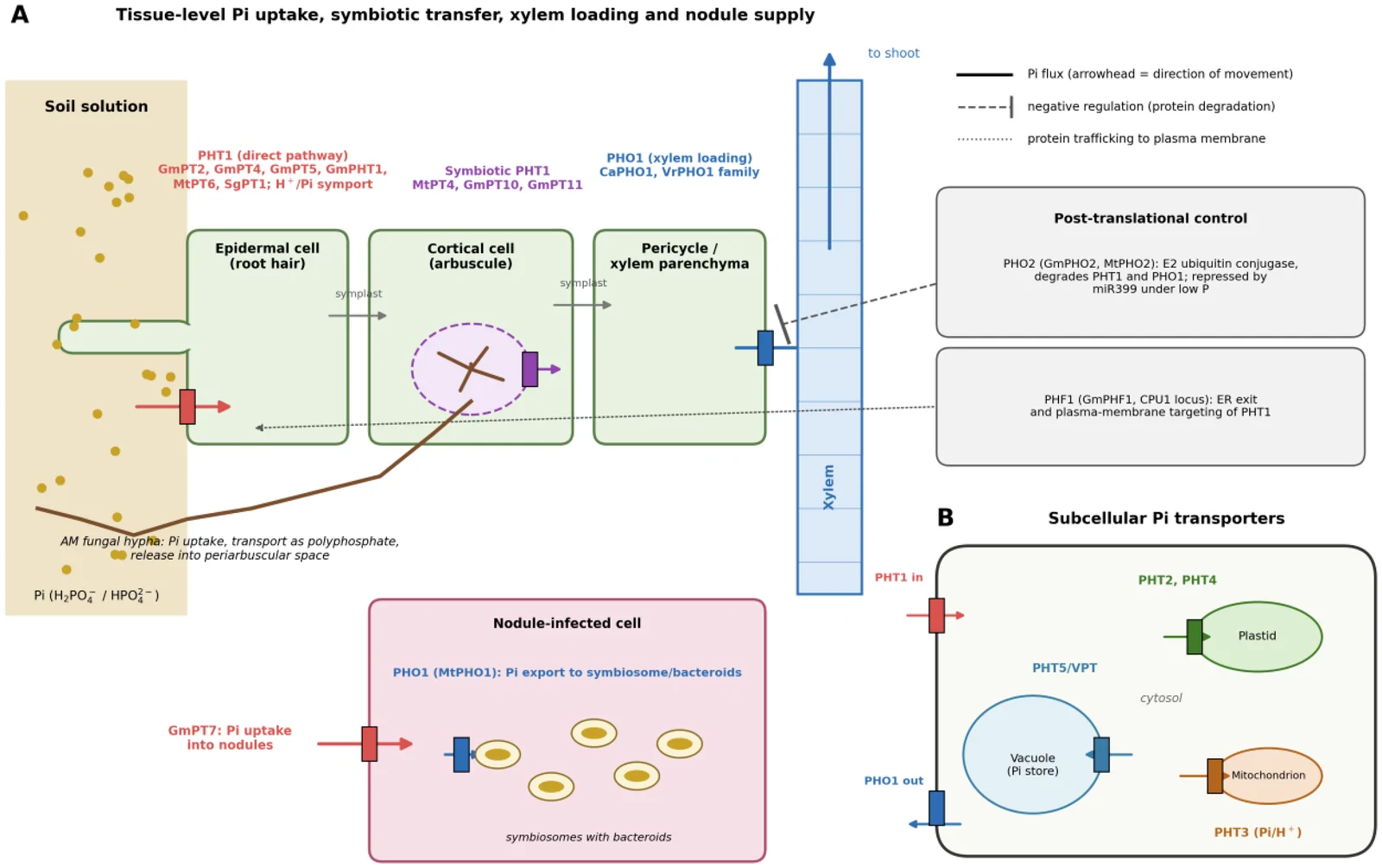
Phosphate transporters in legumes: cellular localization and direction of Pi transport. **(A)** Tissue level. In the direct pathway, plasma-membrane PHT1 transporters (e.g., GmPT2, GmPT4, GmPT5, GmPHT1, MtPT6, SgPT1) import Pi from the soil solution into epidermal and root-hair cells by H^+^/Pi symport. In the symbiotic pathway, arbuscular mycorrhizal hyphae take up Pi, transport it as polyphosphate and release it into the periarbuscular space, from which mycorrhiza-inducible PHT1 transporters (e.g., MtPT4, GmPT10, GmPT11) import it into cortical cells. After symplastic movement, PHO1 family proteins (e.g., CaPHO1, *V. radiata* PHO1 family) export Pi from pericycle and xylem parenchyma cells into the xylem for transport to the shoot. In nodules, GmPT7 supplies Pi to nodules and PHO1 (MtPHO1) exports Pi from infected cells towards the symbiosomes and bacteroids. PHO2 (GmPHO2, MtPHO2) promotes the degradation of PHT1 and PHO1 (negative regulation), whereas PHF1 (GmPHF1) facilitates the trafficking of PHT1 to the plasma membrane. **(B)** Subcellular level. PHT1 imports and PHO1 exports Pi across the plasma membrane; PHT2 and PHT4 transport Pi into plastids (PHT4 also in the Golgi apparatus), PHT3 into mitochondria, and PHT5/VPT into the vacuole for storage. Arrowheads indicate the direction of Pi movement.

**Figure 5:**
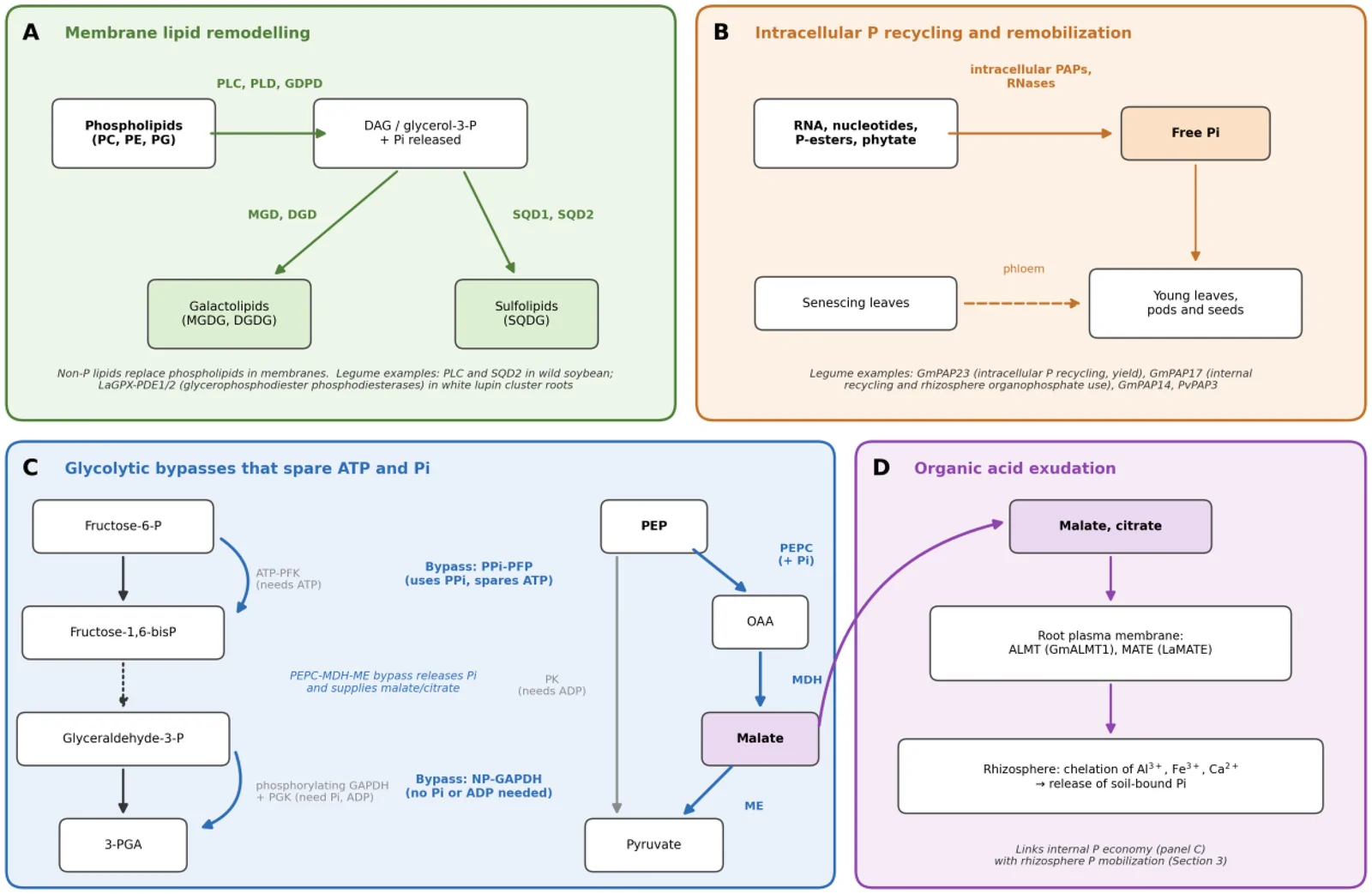
P-saving and P-recycling metabolic pathways that increase internal PUtE in legumes. **(A)** Membrane lipid remodeling: phospholipids are hydrolyzed by phospholipases (PLC, PLD) and glycerophosphodiester phosphodiesterases (GDPD), releasing Pi, and are replaced by galactolipids (MGDG, DGDG) and sulfolipids (SQDG). **(B)** Intracellular P recycling and remobilization: intracellular purple acid phosphatases (PAPs) and ribonucleases release Pi from organic P compounds, and Pi is remobilized via the phloem from senescing leaves to young leaves, pods and seeds. **(C)** Glycolytic bypasses: pyrophosphate-dependent phosphofructokinase (PPi-PFP), non-phosphorylating glyceraldehyde-3-phosphate dehydrogenase (NP-GAPDH) and the phosphoenolpyruvate carboxylase (PEPC)–malate dehydrogenase (MDH)–malic enzyme (ME) route circumvent reactions that require ATP, ADP or Pi (gray). **(D)** Malate and citrate produced through these bypasses are exuded via ALMT and MATE transporters and release soil-bound Pi by chelating Al^3+^, Fe^3+^ and Ca^2+^. Legume genes discussed in the text are indicated.

### 7.1 Key gene families involved in P sensing, signaling, and root development

At the core of P-starvation signaling in plants is a conserved module formed by PHOSPHATE STARVATION RESPONSE (PHR) transcription factors and SPX-domain proteins. When cellular Pi is sufficient, inositol pyrophosphate signaling molecules accumulate and bind SPX proteins, which then interact with PHR and prevent it from activating its targets; under P deficiency, these signaling molecules decline, PHR is released and binds P1BS *cis*-elements in the promoters of P-starvation-induced genes (Puga et al., 2014; Wild et al., 2016). PHR targets include phosphate transporters, purple acid phosphatases, genes of membrane lipid remodeling and the microRNA miR399, which moves from shoot to root and suppresses *PHO2*, an E2 ubiquitin-conjugating enzyme that promotes the degradation of phosphate transporter 1 (PHT1) and PHO1 transporters (Valdés-López et al., 2008; Huertas et al., 2023). This framework, established largely in *Arabidopsis* and rice (*Oryza sativa*), is conserved in legumes and provides a way of organizing the many legume genes reported to affect PUE: most act either as components of this signaling module or as its downstream effectors in root development, P mobilization and P transport (**Figure 3**).

P-starvation signaling is regulated by transcriptional networks involving PHR and SPX proteins. In soybean, *GmPHR1* functions as a MYB-type transcription factor involved in the phosphate-starvation signaling pathway. Under P-deficient conditions, *GmPHR1* regulates downstream P-responsive genes, including genes associated with phosphate acquisition, P recycling and P homeostasis (Lu et al., 2020). *GmPHR25* is another member of the PHR family involved in phosphate homeostasis in soybean. It participates in the P-signaling network and regulates the expression of several P-starvation-responsive genes (Xue et al., 2017).

SPX-domain proteins are also important components of P homeostasis and signaling. In *P. vulgaris*, *PvSPX1* participates in the phosphate-starvation response and regulates the expression of P-responsive genes (Yao et al., 2014b). In soybean, SPX-related genes are associated with the regulation of P concentration and the expression of downstream P-responsive genes (Yao et al., 2014a). These observations indicate that P-signaling pathways coordinate P acquisition, recycling and internal homeostasis rather than functioning through individual genes independently.

Downstream of PHR, the miR399–PHO2 module controls how much Pi is taken up and translocated to the shoot. In common bean, PvPHR1 and PvmiR399 are essential components of P-deficiency signaling in roots (Valdés-López et al., 2008). In *M. truncatula*, the *PHO2* genes have distinct roles in P homeostasis and symbiotic nitrogen fixation (Huertas et al., 2023), and alfalfa (*Medicago sativa*) *pho2* mutants hyperaccumulate phosphate (Miller et al., 2022). These results identify PHO2 as a potential target for increasing P uptake, but they also show that its loss of function can cause Pi over-accumulation, indicating that improving PUE requires fine-tuning rather than simple maximization of P-starvation signaling.

Downstream of these signaling components, several genes control root growth under low P. Root development and architecture are important components of P acquisition because they determine the soil volume explored by the root system. In *G. max*, several genes associated with root development are induced under low-P conditions. *GmEXPB2* encodes a cell-wall-localized β-expansin protein that promotes primary root elongation by modifying cell-wall properties and increasing the size and number of cortical cells in the root meristematic and elongation zones. These changes in root architecture can increase the capacity of plants to acquire P and consequently improve PAE and PUE (Zhou et al., 2014). Similarly, *GmEXLB1* encodes an expansin-like protein that contributes to root elongation and secondary root development, thereby increasing root surface area and improving soil exploration for P acquisition (Kong et al., 2019).

*GmETO1* is involved in ethylene signaling and contributes to root development and P acquisition. Its induction under low-P conditions regulates downstream genes associated with root development and P acquisition, including *GmACP1/2*, *GmPht1/4*, *Expansin-A7*, and *GmRDP1.* These responses promote root proliferation and improve P acquisition under P-deficient conditions (Zhang et al., 2020).

Purple acid phosphatases (PAPs) also contribute to P utilization by releasing Pi from organic P compounds. In soybean, genes such as *GmPAP7a*, *GmPAP7b*, *GmPAP14*, and *GmACP1* have been associated with organic P utilization and improved P availability (Zhu et al., 2020b). *GmPAP17* is predominantly expressed in roots and contributes to rhizosphere organophosphate mobilization and internal P recycling, supporting PUE (Xu et al., 2022).

Other genes contribute to PUE through regulation of root growth and rhizosphere processes. *In L. albus*, genes associated with citrate and malate transport and cluster-root development contribute to P mobilization and acquisition. *LaMATE1/3*, *LaABCG29*, *LaABCG37*, and *LaGPX-PDE1/2* are associated with rhizosphere P mobilization, root-hair development and cluster-root functioning, whereas *LaCEP1* acts as a negative regulator of cluster-root formation and P acquisition (Xu et al., 2020; Aslam et al., 2021; Zhou et al., 2019). In *S. guianensis*, *SgEXPB1* has been associated with improved root elongation and P uptake under P-deficient conditions (Wang et al., 2023b) (**Table 3**).

Overall, the genetic regulation of PUE involves interconnected pathways controlling root architecture, P-starvation signaling, organic P mobilization, P recycling, and rhizosphere adaptation. The contribution of individual genes may vary with genotype and P availability, indicating that PUE is a complex trait controlled by multiple interacting genetic mechanisms.

### 7.2 Phosphate transporters: cellular localization and direction of Pi transport

Once Pi has been mobilized in the rhizosphere, its movement into and within the plant depends on several families of phosphate transporters that differ in their membrane localization and in the direction in which they move Pi (**Figure 4**). PHT1 proteins are high-affinity H^+^/Pi symporters in the plasma membrane that import Pi from the soil solution or, in mycorrhizal roots, from the periarbuscular space. PHO1 proteins export Pi out of cells, for example from root pericycle and xylem parenchyma cells into the xylem. PHT2, PHT3, and PHT4 transporters move Pi across the membranes of plastids, mitochondria, and the Golgi apparatus, and vacuolar PHT5 (VPT) transporters load excess Pi into the vacuole, which acts as a buffer that can later be remobilized (Hamburger et al., 2002; Liu et al., 2016).

Genes of the *PHT1* family play a crucial role in the absorption, transport, and redistribution of phosphate inside plants (An et al., 2023; Jia et al., 2025; Singh et al., 2025b). Specific *PHT1* transporters, such as *LbPT7*, which promoted Pi uptake when overexpressed in *Nicotiana tabacum* (Li et al., 2024), and *SgPT1* in *S. guianensis* (An et al., 2023), have been identified to play an important role in phosphate uptake. For instance, overexpression of *LbPT7* can improve phosphate uptake by plants and can also influence phosphate acquisition by AM fungi under high phosphate levels (Li et al., 2024). In legumes, *PHT1* transporters are expressed at the symbiotic interface inside the arbuscules, where they regulate the transfer of phosphate from the fungus into the plant. Members of the *PHO1* gene family participate in the loading of phosphate into the xylem for long-distance transport within the plant (Nguyen et al., 2020; Jiang et al., 2025). In *M. truncatula*, members of the *PHO1* family are essential for transporting phosphate from infected nodule cells to bacteroids, emphasizing their importance in maintaining the P requirement of the nitrogen fixation process (Nguyen et al., 2020). For instance, studies in *V. radiata* have identified members of the *PHO1* family and investigated their response to P deficiency, and associated them with P stress tolerance and yield performance (Jiang et al., 2025).

In soybean, several high-affinity phosphate transporters, including *GmPT2*, *GmPT4*, *GmPT5*, *GmPT7*, and *GmPHT1*, have been identified. *GmPT4* functions in phosphate uptake and homeostasis and is localized to the plasma membrane of root epidermal cells, where it directly facilitates phosphate uptake from the soil. Overexpression of *GmPT4* increases P uptake, plant growth, biomass accumulation, and P-acquisition efficiency (Guo et al., 2022). *GmPT7* is associated with root- and nodule-mediated P acquisition and is induced under low-P conditions, contributing to phosphate transport to roots and nodules and supporting symbiotic nitrogen fixation (Chen et al., 2019). *GmPT8*, *GmPT9*, *GmPT10*, and *GmPT11* belong to the PHT1 family of phosphate transporters in soybean. *GmPT10* and *GmPT11* are primarily associated with mycorrhiza-mediated P uptake. Following AM fungal colonization, their increased expression suggests a shift toward the symbiotic phosphate-acquisition pathway. In this pathway, phosphate absorbed by fungal hyphae from the soil is transferred to plant cells through arbuscules, with *GmPT10* and *GmPT11* serving as important transporters at the plant–fungus interface. In contrast, *GmPT8* and *GmPT9* are less AM-specific and are associated with general phosphate transport under non-symbiotic conditions (Cui et al., 2019).

Two conclusions emerge from the transporter studies. First, legumes use distinct sets of PHT1 transporters for the direct (root epidermis) and the symbiotic (periarbuscular membrane) uptake pathways, and the relative contribution of these pathways changes with mycorrhizal colonization (Fellbaum et al., 2014; Cui et al., 2019). Second, nodules require their own Pi supply through transporters such as GmPT7 and PHO1 homologs, which directly links P transport to nitrogen fixation (Chen et al., 2019; Nguyen et al., 2020; Mani et al., 2024). Nevertheless, most transporter functions have been validated only by overexpression or hairy-root assays under controlled conditions, and natural allelic variation in transporter-related genes remains largely unexplored for breeding. A notable exception is the soybean *GmPHF1* gene, which facilitates the trafficking of phosphate transporters to the plasma membrane and underlies natural variation in P acquisition (Guo et al., 2022; Li et al., 2022b).

In addition to these particular genes, research also emphasizes the importance of genetic variation in root morphological traits, such as root length, root surface area and specific root length, which are critical for effective P acquisition (Thudi et al., 2021; McLachlan et al., 2020; Sai-Sai et al., 2024). These root traits make plants explore a maximum soil volume and enhance contact with available P.

### 7.3 Transcriptomic and proteomic insights into P-starvation responses

Integrated genomics, transcriptomics, proteomics and metabolomics studies indicate that PUE is a multi-layered trait controlled by coordinated genetic, metabolic and regulatory networks (Xue et al., 2022; Gupta et al., 2023; Sharma et al., 2024; Zalzalah et al., 2025). Transcriptome studies consistently show that P deficiency triggers large-scale reprogramming of gene expression in legumes and that this response is developmentally regulated. In common bean roots, P stress altered the expression of genes involved in P transport, carbon metabolism and lipid remodeling, accompanied by corresponding changes in metabolite levels (Hernández et al., 2007). In the P-efficient soybean cultivar Heinong 551 grown under severe P limitation, 15,401 differentially expressed genes (DEGs) were detected, 94% of them at early developmental stages; the flowering stage alone accounted for 10,660 DEGs, enriched in photosynthesis, isoflavonoid biosynthesis and membrane and cuticle remodeling and regulated mainly by bHLH, bZIP, WRKY, MYB, and ARF transcription factors (Liu et al., 2025). These data suggest that much of the P-efficiency response is established before pod filling. In barren-tolerant wild soybean, Pi transporters and acid phosphatases (ACP1 and PAPs) were upregulated together with genes of phenolic, lignin and organic acid metabolism, and HSFA6b and MYB61 were identified as candidate regulators (Li et al., 2022a). In pigeonpea, integrated genome and transcriptome analyses linked the P-starvation response to recent tandem expansion of the *PHT1*, *PECP*, *FLA*, and *GAD* gene families (Liu et al., 2022), and in *S. guianensis*, 15 PAP genes were upregulated under Pi deficiency, including the root-secreted *SgPAP10*, which enhances organic-P utilization (Wu et al., 2023). In soybean, the genes most consistently induced under P deficiency encode PHT1 transporters, PAPs, SPX proteins, and enzymes of lipid remodeling (Mo et al., 2022).

Proteomic and phosphoproteomic analyses add a further regulatory layer, because many P-starvation responses are controlled post-translationally, through protein phosphorylation, the trafficking of transporters to the plasma membrane and the secretion of phosphatases, and methods for quantitative phosphoproteomics in legumes are now available (Barua et al., 2020). At the protein level, secreted and intracellular phosphatases such as GmPAP17 contribute both to rhizosphere organophosphate mobilization and to internal P recycling (Section 7.1; Xu et al., 2022). However, proteomic studies on P deficiency in legumes remain far fewer than transcriptomic studies, and transcript and protein abundance are often poorly correlated. Integrating both levels is therefore necessary before candidate genes identified from transcriptome data are prioritized for breeding.

### 7.4 Metabolomic analyses of P-saving metabolic pathways

Metabolomic studies provide the most direct view of how legumes economize on P internally. Under P deficiency, plants activate several P-saving and P-recycling pathways (**Figure 5**; Plaxton and Tran, 2011; Veneklaas et al., 2012). First, membrane phospholipids, which contain a substantial fraction of cellular organic P, are hydrolyzed by phospholipases and glycerophosphodiester phosphodiesterases and replaced by non-phosphorus galactolipids and sulfolipids. In wild soybean, this lipid remodeling involved phospholipase C and the sulfolipid synthase SQD2 (Li et al., 2022b), and in white lupin, glycerophosphodiester phosphodiesterases are required for cluster-root and root hair development under P deficiency (Cheng et al., 2011). Second, intracellular PAPs and ribonucleases release Pi from nucleic acids and other organic P esters, as shown for GmPAP23, which mediates intracellular P recycling and affects yield in soybean (Guo et al., 2025). Third, P-deficient cells use alternative glycolytic enzymes, such as pyrophosphate-dependent phosphofructokinase, non-phosphorylating glyceraldehyde-3-phosphate dehydrogenase and the phosphoenolpyruvate carboxylase–malate dehydrogenase bypass, which reduce the dependence of carbon metabolism on adenylates and Pi (Plaxton and Tran, 2011).

The last of these bypasses also links the internal P economy to rhizosphere P mobilization, because it produces the organic acids that are exuded by roots (*Section 3*). Metabolite profiling consistently shows increases in organic acids, amino acids and sugars under P deficiency (Han et al., 2022; Li et al., 2022b), and differences in malate dehydrogenase transcript abundance and enzyme activity are associated with differential carboxylate efflux among soybean genotypes (Vengavasi et al., 2016; Zhu et al., 2021). In *S. guianensis*, Pi deficiency increased the secretion of tartaric acid, L-cysteine and flavonoids (Wu et al., 2023). Root exudation of citrate and related organic acids remains a key mechanism for mobilizing insoluble soil P (Tiziani et al., 2021; Kamran et al., 2020). A limitation of current legume metabolomic studies is that they are usually performed on few genotypes and at a single time point; analyses that combine metabolite profiles with the measurement of leaf P fractions, as done in maize (Han et al., 2022), would help to identify which metabolic traits distinguish P-efficient from P-inefficient genotypes in a heritable way.

Overall, molecular and multi-omics studies indicate that PUE is regulated through coordinated changes in P transport, sensing, root development, and metabolism. Although several candidate genes and regulatory pathways have been identified, their responses can vary among genotypes and experimental conditions. Therefore, further functional validation and field-level evaluation are needed to confirm their contribution to PUE and their potential application in breeding. Nevertheless, the identification of genes and regulatory networks involved in P acquisition and utilization provides a mechanistic basis for translating molecular discoveries into genomic, marker-assisted, and genome-editing strategies for P-efficient legume breeding.

## 8 Mapping the genetic architecture of PUE: QTL mapping and GWAS

Molecular studies identify genes that can influence P efficiency, but breeding also requires knowing which genomic regions explain the variation present in breeding germplasm. Two complementary approaches have been used: biparental QTL mapping, which has high power to detect loci that segregate between two contrasting parents, and GWAS, which exploits historical recombination in diverse panels to achieve higher mapping resolution (**Table 4**).

**Table 4:** Details of gene/QTLs associated with PUE identified in legumes.

Crop	Linkage group/chromosome	QTL/gene	Population type	Related traits	References
*V. unguiculata*	Chr 9, 10	*Vigun09g030400, Vigun10g08400*	MAGIC	Shoot PUE and whole-plant PUE under low P	Ohlson et al. (2025)
*G. max*	Chr 4, 7, 8, 10, 15	12 QTLs	RIL, Zhonghuang 15 × Niumaohuang	Root dry weight, root fresh weight, total biomass	Zhang et al. (2022)
*G. max*	Chr 3, 13, 15, 19, 20	*GmABCG39*	GWAS (219)	Seed yield, 100-seed weight, plant height, nod number of main shoot	Wang et al. (2022)
*G. max*	9 linkage group	12 QTLs	Zhongdou27 × Jiunong 20, RIL (112)	Plant growth, 100-seed weight	Wu et al. (2020)
*G. max*	Chr 6, 10, 11	*GmSPX-RING1*; 35 significant MTA	GWAS (211)	Shoot dry weight, shoot P concentration	Du et al., 2020
*G. max*	Chr 4, 7, 8, 10, 12	14 Candidate genes	RIL, Zhonghuang 15 × Niumaohuang	Plant height, root biomass, P concentration and P utilization efficiency	Zhang et al. (2022)
*G. max*	LG B2-1, D1b + W, and G	34 QTLs	Nannong 94-156 × Bogao, RIL	Root dry weight, shoot dry weight, total dry weight, P use efficiency	Zhang et al. (2009)
*G. max*	Chr 2, 3, 4, 12, 14, 15, 19	172 QTLs	Bogao × Nannong 94-156, RIL (152)	Shoot dry weight, root dry weight, total dry weight, root to shoot ratio	Li et al. (2016)
*P. vulgaris*	Chr 1, 3, 4, 5, 8, 9, 10, 11	19 QTLs	DOR364 × G19833, RIL (86)	Total acid exudation, root hair density, total root hair length, P uptake	Yan et al. (2004)
*P. vulgaris*	Chr 1, 2, 3, 4, 5, 6, 7, 8, 11	15 QTLs	G19833 × AND696, RIL	Root length density, root surface area, seed P content, P uptake	Cichy et al. (2009)
*C. arietinum*	Ca 1, Ca 7, Ca 4	*Ca1_12310101, Ca7_33808891, and Ca4_8269508*	GWAS (206)	P use efficiency, shoot P content, shoot dry weight	Thudi et al. (2021)
*V. radiata*	LG 8, 7, 4	*VRADI11G08340, VRADI04G10750, and VRADI01G05520*	GWAS (206)	Total root length, total root surface, total root tip, RDW, SDW, root shoot ratio	Reddy et al. (2020)
*C. arietinum*	LG 1, 4, 5, 6, 7	*Ca_01588, E3 SUMO-protein ligase-SIZI, MYB-related protein 306*	GWAS (100)	Root hair length	Kohli et al. (2020)

### 8.1 QTL mapping of P-efficiency traits

Identifying QTLs for PUE is a priority given the finite nature of P resources (Jha et al., 2023). Ohlson et al. (2025) identified three significant SNPs controlling PUE in *V. unguiculata* by GWAS of a multi-parent advanced generation inter-cross (MAGIC) population. Two SNPs located on chromosomes Vu09 and Vu10 were associated with whole-plant and shoot PUE, while one SNP located on Vu03 was associated with root system PUE. Candidate gene analysis indicated that the Vu09 locus is associated with genes encoding adenosylcobalamin biosynthesis and PAP involved in the mobilization of P, the Vu10 locus associated with leucoanthocyanidin dioxygenase involved in stress tolerance, and the Vu03 locus associated with peroxidase and transcriptional regulator genes influencing root development and adaptation under P-deficiency conditions.

In 2009, Zhang and co-workers conducted QTL mapping for low-P tolerance in *G. max* using 152 recombinant inbred lines (RILs) derived from a cross between the P-tolerant genotype Nannong94–156 and the P-sensitive genotype Bogao. Three major QTL clusters were detected on linkage groups D1b + W, B2-1, and G, associated with P-efficiency traits. The Satt274–Satt703 marker interval on linkage group D1b + W was strongly associated with root biomass, shoot biomass, total biomass, PUE and PAE, indicating a stable genomic region useful for breeding soybean varieties tolerant to low-P conditions. Li et al. (2016) used the same population to understand genetic variation in photosynthesis and PUE-related traits in *G. max*. They identified a total of 172 QTLs for photosynthetic and P-efficiency-related traits. These QTLs were classified into 29 consensus genomic regions; among the identified regions, 12 genomic regions (approximately 41%) were associated with both photosynthetic traits and P efficiency, suggesting a significant genetic relationship between these traits. Three major QTLs, q15-2, q14-2, and q19-2, were consistently identified for P utilization and photosynthetic performance under low-P conditions. Candidate gene analysis within major QTL regions revealed genes involved in photosynthesis and P metabolism, including a PAP gene, *Glyma.19G193900*, which exhibits significant expression under limited-P conditions and was proposed as a major regulator of both traits. Zhang et al. (2022), developed linkage mapping using a 270-line RIL population of G*. max* validated 12 QTLs that co-localized with GWAS-identified loci, confirming the reliability of key genomic regions controlling P utilization. In addition, they identified 14 candidate genes near stable SNP/QTL regions, including PAPs, MYB transcription factors, sugar transporters, and HSP20-like chaperones. Transcriptome analysis and qRT-PCR confirmed that genes such as *Glyma.10g176400*, *Glyma.04g101900*, and *Glyma.12g114200* were strongly induced under P-deficiency conditions, highlighting their functional role in P utilization.

Yan et al. (2004) used a RIL population of *P. vulgaris* derived from a cross between DOR364 (P-inefficient) and G19833 (P-efficient). They identified 19 QTLs related to root hair traits, acid exudation, and P uptake across eight linkage groups. Among these, three major QTLs, Hex4.1, Tae4.1 and Hex10.1, were significantly associated with P uptake under field conditions (**Table 4**).

Comparison of these studies shows that QTLs for P efficiency are numerous and mostly of small to moderate effect, that many co-localize with QTLs for biomass, root traits or photosynthesis, and that relatively few have been validated in independent populations or environments. The co-localization of P-efficiency and photosynthesis QTLs in soybean (Li et al., 2016) and the recurrence of PAP genes among candidate genes in independent studies (Li et al., 2016; Zhang et al., 2022; Ohlson et al., 2025) are among the most consistent findings. By contrast, QTL positions for similar traits often differ between populations and P treatments, reflecting different parental combinations, phenotyping methods and strong QTL × environment interactions.

### 8.2 GWAS-based identification of genes

GWAS utilizes natural genetic variation within diverse germplasm collections to identify associations between single nucleotide polymorphisms (SNPs) or insertion and deletions (InDels) and phenotypic traits (Hong et al., 2024). This approach has been widely applied across various legume species to understand the genetic basis of PUE (Yang et al., 2020, Yang et al., 2021b; Du et al., 2020; Wang et al., 2022; Zhang et al., 2022). For instance, one study identified two novel loci for photosynthetic traits related to P efficiency (Yang et al., 2020). Another comprehensive study on soybean used chlorophyll fluorescence parameters to identify P-efficiency QTLs and predict candidate genes through GWAS and RNA-seq, highlighting the strong relationship between photosynthesis and P efficiency (Yang et al., 2021b).

A notable study in *G. max* identified a strong association signal at the component of phosphorus uptake 1 (CPU1) locus, revealing *GmPHF1*, a SEC12-like gene, as the causal gene responsible for variation in PUE and P acquisition among soybean genotypes (Guo et al., 2022). Another GWAS in soybean, using the NJAU 355 K SoySNP array, identified SNPs significantly associated with P-related traits and predicted the candidate gene *GmSPX-RING1* responsible for P efficiency (Du et al., 2020). More recently, studies have focused on P-efficiency-related traits at maturity, as approximately 90% of P uptake occurs during the reproductive stage (Wang et al., 2022). A GWAS evaluated six P-efficiency-related traits at maturity in 219 soybean accessions, contributing to understanding low-P tolerance mechanisms in later growth stages (Wang et al., 2022). Additionally, a study integrating GWAS and linkage mapping on 11 P-utilization-related traits in 281 soybean germplasm accessions and a RIL population revealed important QTLs and candidate genes, underscoring the value of combining different genetic mapping strategies (Zhang et al., 2022). In chickpea, a genome-wide association mapping approach identified novel genes and genetic loci associated with root architecture, PAE and PUE, providing strategies to reduce P-fertilizer application (Thudi et al., 2021). Similarly, mung bean studies have genetically dissected PUpE and PUtE traits using GWAS on an association mapping panel of 120 genotypes (Reddy et al., 2021a). Another GWAS in mung bean, using a mini-core collection, examined the genetic basis of variation in important traits, including those potentially related to nutrient efficiency (Sokolkova et al., 2020).

The success of GWAS for PUE relies on several factors, including diverse germplasm collections, high-density genotyping platforms (e.g., SNP arrays), and accurate phenotyping of P-related traits under varying P environments (Liang et al., 2025). Phenotypic analysis under P deficiency has shown significant variation in traits such as root length, shoot dry weight, and chlorophyll content, which are critical for PUE (Wei et al., 2025; Neji et al., 2019). For example, in cotton seedlings, phenotypic analysis under low-P stress revealed substantial variation, with low-P- shoot dry weight (low-P-SDW) showing a high coefficient of variation of 33.69% (Wei et al., 2025). Integration with other genomic and transcriptomic approaches, such as RNA sequencing (RNA-seq), enhances the findings from GWAS by providing functional context for identified genes. This multi-omics strategy allows for a more comprehensive understanding of the genetic mechanisms underlying PUE (Liang et al., 2025). The identified candidate genes often include those involved in P transport, signal transduction, and metabolic pathways. For instance, the *GmSPX-RING1* gene in soybean is involved in P signaling (Du et al., 2020), while SEC12-like genes such as *GmPHF1* are involved in protein transport and potentially P acquisition (Guo et al., 2022).

The application of GWAS findings extends beyond the identification of genes. It informs the development of genomic selection strategies and marker-assisted breeding for increased PUE (Wang et al., 2022). However, challenges remain, including the need for accurate phenotyping across diverse environments and the interpretation of complex genetic interactions, especially gene–environment interactions (Liang et al., 2025; Yang et al., 2020; Roller and Wurschum, 2024). Across legumes, the most robust GWAS results are those in which associations were confirmed by linkage mapping, expression analysis or functional validation, as for *GmPHF1* at the CPU1 locus and *GmSPX-RING1* in soybean (Guo et al., 2022; Du et al., 2020); most other GWAS loci for PUE remain candidate associations detected in single panels and environments.

## 9 Translational strategies for developing P-efficient legumes

*Sections 2–8* identify the physiological traits, microbial partners, genes and genomic regions that contribute to PUE. This section considers how these components can be combined in breeding programs. Because these strategies draw on all of the preceding sections, rather than on QTL and GWAS results alone, they are presented here as a separate section.

### 9.1 Functional genomics and genome editing

Functional genomic methodologies provided valuable insights regarding the functional roles of specific genes in the enhancement of PUE across various grain legumes (Xue et al., 2017). In an effort to reveal the functional role of *PHR*, a well-established gene involved in the regulation of phosphate uptake, Xue et al. (2017) demonstrated that the overexpression of the *GmPHR25* gene, a member of the *GmPHR* gene family, resulted in an enhanced concentration of Pi in transgenic soybean plants cultivated under standard conditions. Furthermore, the overexpression of *GmPHR25* was observed to enhance the expression of five Pi-starvation-related genes, including *GmHAD1-2*, *GmEXPB2*, *GmSPX5*, *GmPAP21*, and *GmPAP14*, thereby indicating that *GmPHR25* assumes a pivotal role within the P-signaling network of soybean. In a similar vein, the genetically engineered overexpression of the soybean β-expansin gene *GmEXPB2*, recognized for its significant involvement in regulating the root system architecture response to P starvation (Guo et al., 2011). Among the various transcription factors involved in the regulation of PUE, the role of the WRKY TF family is notably prominent. Li et al. (2021b) illustrated that transgenic soybean exhibiting overexpression of *GmWRKY46* demonstrated improved development of hairy roots, consequently leading to increased plant growth and enhanced P uptake. Similarly, investigating the contribution of the *6-phosphogluconate dehydrogenase* (*6PGDH*) gene to low-P tolerance, Li et al (2021) identified that transgenic overexpression of the *Gm6PGDH1* gene facilitated increased PUE by promoting root development, altering antioxidant mechanisms, and enhancing P uptake in soybean under P-deficient conditions. To elucidate the regulatory function of the *SPX1* domain-containing protein-encoding gene involved in the P-signaling network, Yao et al. (2014b) established that overexpression of *PvSPX1* resulted in increased expression of several Pi-starvation-related genes such as *PvPHT2*, *PvPT1*, *PvPAP1*, and *Pv4*, which are crucial for P regulation in *P. vulgaris*. In a comparable study, the overexpression of *GmSPX3* led to enhanced P concentrations in both shoots and roots of soybean, while enhancing the expression of seven Pi-starvation genes (*GmIPS1*, *GmRNS1*, *GmPT7*, *GmPT2*, *GmALMT1*, *GmHAD1-6*, and *GmPAP21*) within hairy roots (Yao et al., 2014a). The ATP-binding cassette (ABC) transporter G (ABCG) subfamily genes are integral to the translocation of biomolecules across cellular membranes (Aslam et al., 2021b). Among various *ABCG* genes, the transgenic overexpression of the *L.albABGC29* gene resulted in increased P accumulation in the cluster roots of white lupin (*L. albus*), thereby enhancing plant growth under conditions of P deficiency (Aslam et al., 2021). Soybean also contains genes that negatively influence PUE (Zhang et al., 2016; Du et al., 2020). In an RNA interference (RNAi) approach, the suppression of *GmSPX-RING1* in soybean under both P-sufficient and P-deficient conditions significantly enhanced P concentrations in hairy roots relative to the control (Du et al., 2020). In contrast, the transgenic overexpression of *GmSPX-RING1* resulted in reduced P concentrations in soybean hairy roots in the P-sufficient environment compared to the control (Du et al., 2020), thus indicating the negative regulatory role of *GmSPX-RING1* in PUE within soybean. CRISPR/Cas9-based genome editing has emerged as a prominent functional genomics methodology for enhancing the functions of genes associated with agricultural traits and for the precise enhancement of such traits. Evidence supporting the improvement of PUE through the manipulation of various P-uptake genes has been documented in both tomato (Zhao et al., 2023) and soybean (Guo et al., 2022). Specifically, the knockout of the *SlPHO1.1* gene utilizing genome editing techniques revealed a strong Pi-starvation response in tomato (Zhao et al., 2023). CRISPR/Cas9-mediated genetic modifications of *phr1*, *phr2*, and *phr3* rice mutants exhibited altered growth patterns in response to divergent P-stress conditions (Guo et al., 2015). The application of CRISPR/Cas9 technology in the editing of the MYB1 transcription factor significantly improved P uptake and accumulation. Similarly, CRISPR/Cas9-mediated knockout of the *PHO2* gene in *M. sativa* led to Pi hyperaccumulation, confirming its role as a negative regulator of P homeostasis (Miller et al., 2022). In the context of soybean, Guo et al. (2022) demonstrated that the overexpression of *GmPT4* within hairy root systems resulted in improved growth performance and increased P uptake in soybean. The targeted knockout of the *GmPT4* gene, which encodes a P transporter, through CRISPR/Cas9 technology resulted in reduced P uptake in soybean root systems. Consequently, the integration of diverse omics methodologies, innovative breeding technologies, and agronomic management strategies is anticipated to promote the development of grain legume crops characterized by enhanced PUE (**Figure 6**).

**Figure 6:**
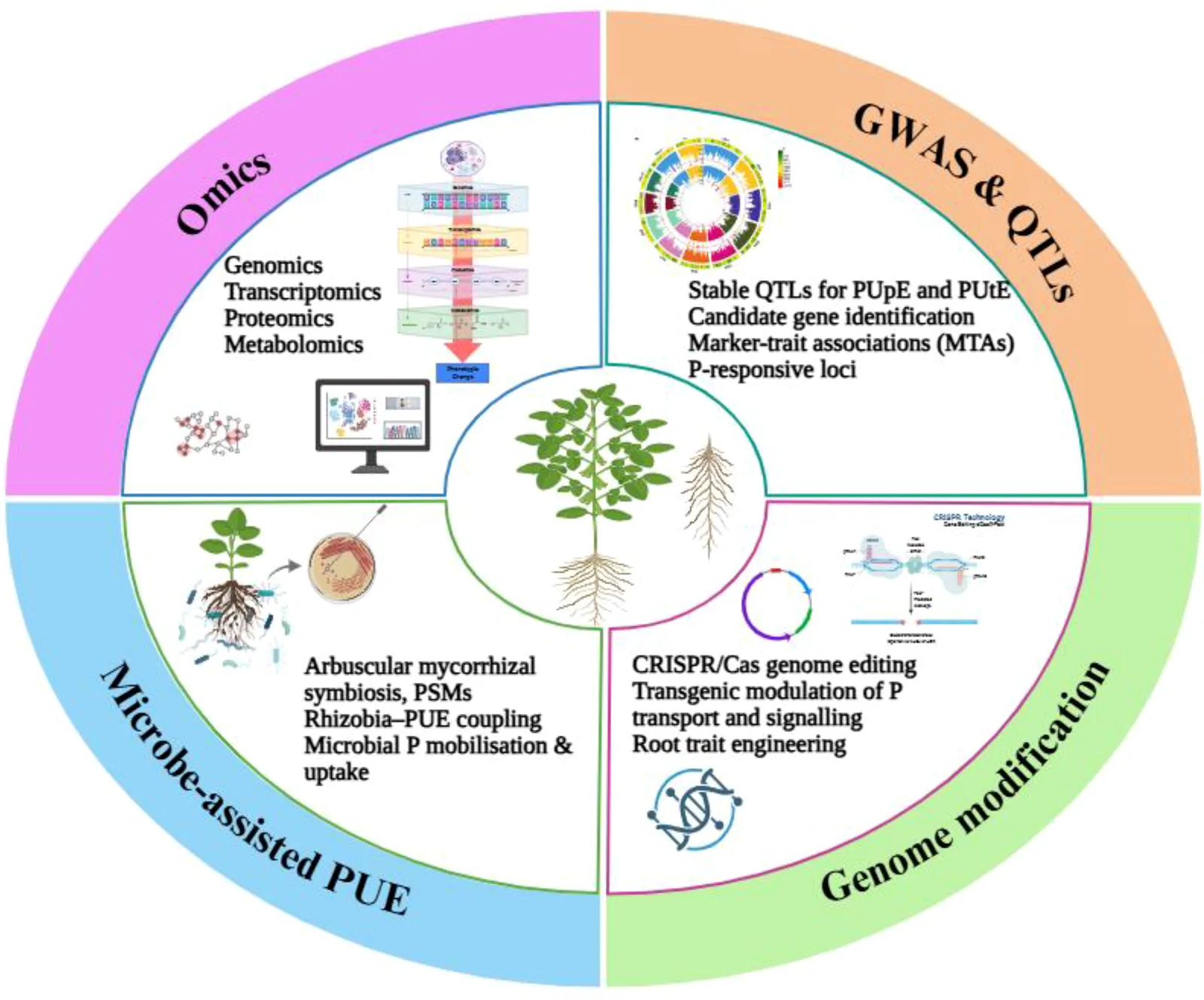
Different approaches for improvement of PUE in legumes.

Two caveats limit the direct use of these results in breeding. Most functional studies relied on constitutive overexpression in soybean hairy roots or heterologous systems under controlled conditions, so their effects on whole-plant PUE and grain yield in the field remain largely unknown. In addition, several genes have pleiotropic effects: overexpressing positive regulators of P-starvation signaling, or knocking out negative regulators such as *PHO2* and *GmSPX-RING1*, can increase P uptake but may also cause excessive Pi accumulation when soil P is adequate (Du et al., 2020; Miller et al., 2022). Precise editing of promoter or upstream open reading frame (uORF) regions, which can fine-tune rather than abolish gene expression, may help to avoid such trade-offs; the natural uORF variant underlying P-acquisition diversity in soybean illustrates the potential of this type of variation (Guo et al., 2022).

### 9.2 Genomic selection and trait-based breeding

Because PUE is controlled by many loci of small effect, marker-assisted selection for individual QTLs is likely to capture only part of the genetic variation. Genomic selection, in which genome-wide markers are used to predict breeding values, is better suited to such traits and has been proposed for improving PUE in grain legumes (Jha et al., 2023), although published applications to P efficiency in legumes are still scarce. In the meantime, combining a small number of validated loci (e.g., the CPU1 locus in soybean) with selection for inexpensive proxy traits can accelerate progress. For example, foliar manganese concentration reflects the release of rhizosheath carboxylates and has been used to screen a large set of chickpea germplasm for this P-mobilizing strategy (Pang et al., 2018b). Multi-environment testing under contrasting P supply will be needed to confirm that such selection improves grain yield per unit of applied P.

### 9.3 Rhizosphere engineering and microbe-assisted breeding

Rhizospheric engineering is the purposeful manipulation of the plant–soil–microbe interactions in the rhizosphere to improve nutrient uptake, plant growth, and stress tolerance (Sadiq et al., 2025). A comprehensive analysis of soil P fractions, microbial biomass, phosphatase activities, and key P-cycling genes, including pqqC, phoC, phoD, and phnK, revealed that legumes significantly increased total and available P in rhizosphere soils compared with non-legumes. Legumes increased both labile and non-labile Pi pools, particularly labile-P, NaHCO_3_-Pi, and residual-P fractions. Higher P availability was associated with greater abundance of functional genes that improved soil pH regulation and phosphatase activities, promoting P solubilization and mineralization. Overall, legumes play a key role in regulating short- and long-term soil P dynamics, supporting vegetation restoration in nutrient-limited calcareous karst soils (Zhu et al., 2025).

For breeding, rhizosphere engineering can be approached from both the microbial and the plant side. On the microbial side, inoculation with phosphate-solubilizing bacteria and AM fungi, alone or together with rhizobia, can improve P uptake and nitrogen fixation in legumes, but field responses are inconsistent because introduced strains often fail to establish in competition with the native microbiota (Bargaz et al., 2021; Janati et al., 2021). On the plant side, host genotypes differ in their ability to recruit and benefit from P-mobilizing microbes, and host genes that control symbiotic signaling, such as those of the common symbiosis signaling pathway and strigolactone biosynthesis (*Section 4*), are potential targets for microbe-assisted breeding. Selecting legume genotypes for responsiveness to beneficial microbes under low-P conditions is therefore a promising, but still largely untested, strategy that would integrate breeding with inoculant development.

## 10 Critical synthesis: consistent findings, discrepancies, and knowledge gaps

Overall, the evidence indicates that PUE in food legumes is controlled by the coordinated contribution of P acquisition, internal P utilization, root system architecture, rhizosphere processes, microbial interactions, and P-responsive regulatory mechanisms. While several responses, such as enhanced root development, P mobilization, and increased expression of P transporters, are commonly observed under P deficiency, their contribution to PUE varies among genotypes and environments. A major knowledge gap is the limited integration of physiological, genetic, molecular, microbial, and agronomic approaches. Future research should therefore focus on identifying stable traits and functionally validated genes and evaluating P-efficient genotypes across contrasting soil P conditions and field environments. Such an integrated approach will help distinguish traits that merely respond to P deficiency from those that directly contribute to improved productivity and PUE.

*Consistent findings.* Several conclusions are supported by multiple studies and species. (i) Many legumes, notably white lupin and chickpea, rely strongly on carboxylate and phosphatase exudation and on symbiotic partners for P acquisition, and nodulated legumes have a high internal P requirement for nitrogen fixation (Lambers et al., 2013; Qin et al., 2012; Pang et al., 2018b). (ii) Substantial genetic variation, often with high heritability for root traits, exists for P-efficiency traits in all food legumes examined (*Section 6*). (iii) The PHR–SPX–miR399–PHO2 signaling module and its targets, particularly PHT1 transporters and PAPs, are conserved regulators of P acquisition and recycling in legumes, and PAP genes recur as candidate genes in independent QTL and GWAS studies (*Sections 7, 8*). (iv) Root hairs are among the most cost-effective P-acquisition traits (*Section 5*).

*Discrepancies.* The literature also contains several discrepancies. Greater root growth or carboxylate exudation increases P uptake in some studies but is not associated with higher biomass or yield in others, reflecting the carbon costs of these traits and their dependence on soil properties (*Sections 3, 5*). Genotype rankings for PUE change with P supply, water availability, and developmental stage, and seedling-stage screens do not necessarily predict performance at maturity (*Section 6*). QTL positions for similar traits often differ between populations and environments, and microbial inoculants that improve P nutrition under controlled conditions frequently give variable results in the field (*Sections 4, 8, and 9*). A further source of apparent disagreement is the inconsistent use of the term PUE itself, which is variously calculated as biomass or yield per unit of P uptake, per unit of P applied, or as relative performance under low P.

*Knowledge gaps.* The main knowledge gaps are: (i) the lack of field-validated evidence that individual genes or QTLs improve grain yield per unit of P in legumes; (ii) limited understanding of internal PUtE, including P remobilization during grain filling and the regulation of seed P and phytate concentration; (iii) insufficient quantification of the carbon costs of acquisition traits and of their interactions with drought and nitrogen fixation; (iv) the scarcity of studies in orphan and tropical food legumes compared with soybean; and (v) limited integration of plant genetic variation with rhizosphere microbial variation. These findings, discrepancies and gaps, together with the corresponding research priorities, are summarized in **Table 5**.

**Table 5:** Summary of consistent findings, discrepancies and knowledge gaps in research on phosphorus-use efficiency (PUE) in food legumes, with suggested research priorities.

Research area	Consistent findings	Discrepancies and uncertainties	Knowledge gaps and research priorities
Root architecture and exudation (*Sections 2, 3*)	Low P increases lateral root and root hair growth and carboxylate/phosphatase exudation; cluster roots in lupins	Greater root growth or exudation does not always increase biomass or yield; effects depend on soil type and P form	Quantify carbon cost per unit P acquired; test trait effects in contrasting soils in the field
Microbial symbioses (*Section 4*)	AM fungi, rhizobia and PSMs improve P acquisition, mainly in low-P soils	Benefits depend on microbial strain, host genotype and soil; inoculant effects often inconsistent in the field	Identify host loci controlling microbial benefit; evaluate inoculant persistence under field conditions
Physiological trade-offs (*Section 5*)	Acquisition traits consume carbon; exudation and mycorrhizal strategies trade off; nodules are strong P sinks	Relative value of PAE versus PUtE depends on production system and co-occurring stresses (e.g., drought)	Define environment-specific ideotypes; study P–N interactions and seed P/phytate trade-offs
Genetic variation (*Section 6*)	Large variation and high heritability for many root traits in all legumes studied	Genotype rankings unstable across P levels, sites and growth stages; inconsistent PUE definitions	Standardize PUE metrics; multi-environment field evaluation to maturity; include orphan legumes
Molecular regulation and transporters (*Sections 7.1, 7.2*)	Conserved PHR–SPX–miR399–PHO2 module; distinct PHT1 sets for direct and symbiotic uptake; PHO1 for xylem and nodule Pi supply	Gene effects vary with genotype and conditions; overexpression may cause Pi toxicity at adequate P	Validate genes in stable transgenic or edited lines in the field; explore natural allelic variation
Multi-omics (*Sections 7.3, 7.4*)	Large-scale, stage-dependent transcriptional reprogramming; lipid remodeling, P recycling and glycolytic bypasses	Poor transcript–protein correlation; most studies use few genotypes and one time point	Integrate omics layers across contrasting genotypes and developmental stages
QTL and GWAS (*Section 8*)	Many small-effect loci; co-localization with biomass and photosynthesis QTLs; recurrent PAP candidate genes	Limited overlap of QTLs across populations and environments; few causal genes validated	Fine-map and validate major loci; develop diagnostic markers for breeding
Translational strategies (*Section 9*)	Genome editing and genomic selection are feasible for PUE-related genes and polygenic variation	Few field-validated edited or genomically selected legume lines for PUE	Edit regulatory regions to fine-tune expression; train genomic prediction models under low P

## 11 Conclusion

Phosphorus scarcity carries a threat to global agricultural security, and even the production of food legumes may be affected. The reviewed strategies range from root architectural modifications, such as increased root length, density, and specialized cluster roots, to physiological adjustments like organic acid exudation and efficient internal P remobilization. At the molecular level, specific genes, including the *PHT1* family transporters, transcription factors like *PHR1*, and enzymes like PAPs, play crucial roles. Furthermore, strong symbiotic interactions with beneficial soil microbes, including AM fungi and rhizobia, are integral to optimizing P uptake. However, these traits carry carbon and metabolic costs, and their value for PUE depends on the target environment. The observed genetic variation within legume crops for these P-efficiency traits provides a robust foundation for developing cultivars better adapted to low-P environments.

Future research should prioritize integrated breeding approaches, combining conventional methods with advanced genomic tools such as GWAS and genomic selection, to accelerate the development of P-efficient legume varieties. A systems biology approach, integrating multi-omics data, is crucial to elucidate the complex regulatory networks governing PUE and PAE. Rhizosphere engineering, focusing on identifying and applying highly effective phosphate-solubilizing microorganisms and mycorrhizal fungi, offers significant potential for enhancing P availability. Gene editing technologies like CRISPR/Cas9 can precisely modify key genes for improved P uptake. The development of high-throughput phenotyping platforms is essential for rapid and accurate assessment of P-efficiency traits. Crucially, field-scale validation across diverse environments is necessary to ensure the practical applicability and farmer adoption of improved varieties and management practices. Finally, understanding the complex interaction between phosphorus and nitrogen metabolism in legumes will be vital for optimizing both nutrient use efficiencies simultaneously, contributing to global food security and reducing reliance on finite phosphate resources.
